# Developmental pattern of grapevine (*Vitis vinifera* L.) berry cuticular wax: Differentiation between epicuticular crystals and underlying wax

**DOI:** 10.1371/journal.pone.0246693

**Published:** 2021-02-19

**Authors:** Katja Arand, Evi Bieler, Markus Dürrenberger, Hanns-Heinz Kassemeyer

**Affiliations:** 1 University of Würzburg, Julius von Sachs Institute for Biosciences, Würzburg, Germany; 2 University of Basel, Swiss Nanoscience Institute—Nano Imaging Lab, Basel, Switzerland; 3 State Institute for Viticulture, Freiburg, Germany; 4 University of Freiburg, Institute of Biology II, Plant Biomechanics Group, Freiburg, Germany; Universidade do Minho, PORTUGAL

## Abstract

The grapevine berry surface is covered by a cuticle consisting of cutin and various lipophilic wax compounds. The latter build the main barrier for transpirational water loss and protect the fruit against environmental factors e.g. pests, mechanical impacts or radiation. The integrety of the fruit surface is one important key factor for post-harvest quality and storage of fruits. Nonetheless, the developmental pattern of cuticular wax was so far only investigated for a very limited number of fruits. Therefore, we performed comparative investigations on the compositional and morphological nature of epicuticular wax crystals and underlying wax during fruit development in *Vitis vinifera*. The main compound oleanolic acid belongs to the pentacyclic triterpenoids, which occur very early in the development in high amounts inside the cuticle. The amount increases until veraison and decreases further during ripening. In general, very-long chain aliphatic (VLCA) compounds are present in much smaller amounts and alcohols and aldehydes follow the same trend during development. In contrast, the amount of fatty acids constantly increases from fruit set to ripening while wax esters only occur in significant amount at veraison and increase further. Wax crystals at the fruit surface are solely composed of VLCAs and the morphology changes during development according to the compositional changes of the VLCA wax compounds. The remarkable compositional differences between epicuticular wax crystals and the underlying wax are important to understand in terms of studying grape-pest interactions or the influence of environmental factors, since only wax crystals directly face the environment.

## Introduction

The outer layer of fleshy fruits, such as berries, fulfils a multiple function as an interface between the fruit containing the seed with the developing embryo and the environment. During the development of the grape berry, the volume increases about 300-fold. In this growth process, the skin of the berries has an important function as a containment of the pericarp since the integrity of the surface must be maintained [1, 2]. The berry skin is the outermost, complex structure of the multi-layered exocarp. It consists of a layer of epidermal cells with a thick outer cell wall coated with the cuticle. The matrix of the cuticle is built by a three-dimensional network preferentially composed of polymerized C16 and C18 hydroxy fatty acids [3]. It is impregnated with aliphatic and cyclic wax components. The aliphatic fraction of grape berry wax comprises homologous series of very-long-chain fatty acids and corresponding derivatives such as aldehydes, primary alcohols, alkyl esters and alkanes [4–6]. The cyclic fraction usually contains pentacyclic triterpenoids particularly of the oleanane and ursane type [5, 7, 8]. Some of the wax crystallizes on the outer surface of the cuticle. These epicuticular wax crystals appear as a glaucous covering on the fruit, making the surface extremely water repellent [9, 10]. The hydrophobic properties of the wax coating have a crucial function as a preformed barrier that protects the berry from uncontrolled water loss and other environmental factors like pathogens and herbivore attack [11, 12]. There is good evidence that the cuticular transpiration barrier is preferentially associated with very-long chain aliphatic wax compounds [3, 13], while triterpenoids contribute to the mechanical strength [14], heat stability [15] or biological activity [16] of the cuticle. During berry development, the cuticle has to overcome different challenges depending on the developmental stage. Due to the very rapid volume expansion, it has to resist strong mechanical stress in order to avoid surface cracking [17]. Therefore, the wax composition is expected to show a dynamic developmental pattern. Changes in the chemical-physical properties during berry development can cause stage-specific susceptibility to diseases and pests [18–20]. In the same way, cultivar-specific differences in cuticular wax can account for different susceptibility among grapevine cultivars [21]. Furthermore, fruit wax plays an important role in post-harvest storage processes and fruit quality [11, 22] because the water loss is related to the physico-chemical properties of the cuticular wax [13, 23, 24]. Here, we performed comparative investigations on the chemical and morphological nature of wax accumulation and the formation of epicuticular wax crystals during fruit development of two prominent *Vitis vinifera* cultivars ‘Müller Thurgau’ and ‘Blauer Spätburgunder’ also known as ‘Pinot noir’. In a novel approach, we differentiated between the epicuticular wax crystals on the outer surface of the cuticle directly facing the atmosphere and interacting pathogens or insects and the underlying wax. To visualize the highly sensitive micro and nanostructure of the epicuticular wax, we used cryo-scanning electron microscopy. This tool enables to detect finest differences in the structure of the wax crystals which would completely disappear during the fixation process for TEM analysis. The scientific results of this work represent significant achievements for targeted breeding of grapevine cultivars with resistance to pests and pathogens and higher berry resilience to environmental changes.

## Material and methods

### Plant material

Grape berries of *Vitis vinifera* L. cv. *Müller Thurgau* (MTH) and cv. *Blauer Spätburgunder* (BSB) (also known as *Pinot noir*) were harvested during the growing season 2015 from directly adjacent plots in the experimental vineyards of the Staatliches Weinbauinstitut, Freiburg im Breisgau, Germany. From each plot three plants were randomly selected, from each of which one cluster was taken. Seven developmental stages from fruit set, were fruits begin to swell (BBCH 71) until berries ripe for harvest (BBCH 89) [25] were investigated (Table 1).

**Table 1:** Overview of the developmental stages of *Vitis vinifera* cv. Müller Thurgau (MTH) and cv. Blauer Spätburgunder (BSB) berries at the specific sampling dates and the respective fruit diameters [25]; Mean(SD).

					Diameter [mm]	
Category		Date	BBCH	Description	MTH	BSB
**Berry formation**	1	23.06.2015	71	Fruits begin to swell	2.9 (0.3)	3.0 (0.4)
	2	29.06.2015	73	Berries groat-sized	5.5 (0.6)	5.4 (0.8)
	3	07.07.2015	75	Berries pea-sized	9.9 (0.7)	9.8 (1.0)
	4	21.07.2015	77	Berries begin to touch	11.9 (0.4)	12.2 (0.4)
**Veraison**	5	04.08.2015	81	Begin of ripening	13,7 (0.4)	13.2 (0.4)
**Ripening**	6	24.08.2015	85	Softening of berries	15.7 (0.2)	15.5 (0.3)
	7	21.09.2015	89	Berries ripe for harvest	16.5 (0.8)	16.6 (0.4)

### Scanning electron microscopy (Cryo-SEM)

Visualization of the three dimensional surface wax structures was carried out with a scanning-electron microscope (Philips XL30 ESEM) equipped with a cryo preparation unit (Alto 2500, Gatan, UK). For this purpose, three intact clusters inserted between the second and third nodes of the shoot were collected early in the morning from the experimental plots. Immediately after sampling, three berries of the same size were removed from the inside of each cluster, from which a slice of the berry skin of approx. 3 mm x 3 mm was excised with a scalpel. The slices were mounted with a low temperature glue on a specimen holder by carefully avoiding touching the waxy surface. Cryofixation was performed with nitrogen slush (< -185°C). The frozen samples were sputtered with 20 nm Au in a high vacuum cryo preparation chamber and examined with a SE detector operating with acceleration voltage of 5–10 kV at high vacuum and -150°C. In this way, the specimens were cryo-fixed, sputtered and ready for analysis within 90 min after sampling in the field. A total of nine samples from each variety were analyzed, the surface structure of each specimen was documented from at least three positions at 8.000x magnification.

### Wax analysis

For the wax analysis, bunches were collected from the experimental plots in the same way as described above. Individual berries of the same developmental stage (Table 1) were carefully cut from the bunches by remaining a small piece of the pedicel to prevent leaching from the wound. The berries were washed with deionized water and air-dried. The fruit surface area was determined from the average of the transverse and longitudinal diameter by assuming a spherical shape of the berries. Five biological replicates (n = 5) were analyzed with six (category 1) to two (category 7) berries per sample, depending on the developmental stage.

### Mechanical removal of epicuticular wax crystals

Small berries up to category 3 were incubated for 5 min in a saturated aqueous sodium chloride solution with ultrasonic to mechanically remove the surface wax. Ethanol (10%) was added to lower the surface tension and ensure a sufficient wetting of the hydrophobic fruit surface. A comparison between treatments with and without ultra-sonic (S1 Fig) showed that epicuticular wax was mechanically grinded off due to the movement of the salt molecules without any chemical extraction, which ensured a separation of the epicuticular wax crystals. After the procedure, berries looked highly polished. As wax crystals became stronger during development, larger berries from category 4 on were carefully rubbed with tissue until they appeared shiny. The tissue and the aqueous solutions were blended with n-tetracosane as internal standard and extracted three-fold with chloroform.

### Chemical wax extraction

After removal of epicuticular wax crystals, berries were extracted with chloroform for 5 min in ultra-sonic. Extracts were washed three times with deionized water to remove hydrophilic organic compounds like sugars. As was described previously, the grape berry wax consists of two fractions, the cyclic triterpenoids and a mixture of long chain aliphatics [26] which were separated with different solvents, to prevent overlying peaks in the gas chromatographic analyzing procedure (S2 Fig). After evaporation to dryness under nitrogen, the residue was dispensed three times in methanol to dissolve the more polar triterpenoid wax fraction. The methanol extracts were filtered and the remaining residue containing the more apolar, aliphatic wax components was dissolved in chloroform. N-tetracosane was added to both fractions as internal standard.

### Analytical procedure

All wax samples were evaporated to dryness and hydroxyl-containing compounds were transformed into their corresponding trimethylsilyl derivatives by incubating in bis-*N*,*O*-(trimethylsilyl)trifluoroacetamide (BSTFA, Macherey-Nagel) in pyridine (Merck) for 30 min at 70° C. The qualitative composition of the wax and the quantity were determined by temperature-controlled capillary gas chromatography and mass spectrometry or flame ionization detection as described earlier [24]. Single compounds were quantified against the internal standard and the amount was normalized to the surface area (μg cm^-2^). The total amount of wax was calculated by summing up all fractions. Methanol and chloroform fractions were added up and compared to the crystal fraction obtained by mechanical removal.

## Results

### During berry development the morphology of the epicuticular waxes alters

**Fig 1:**
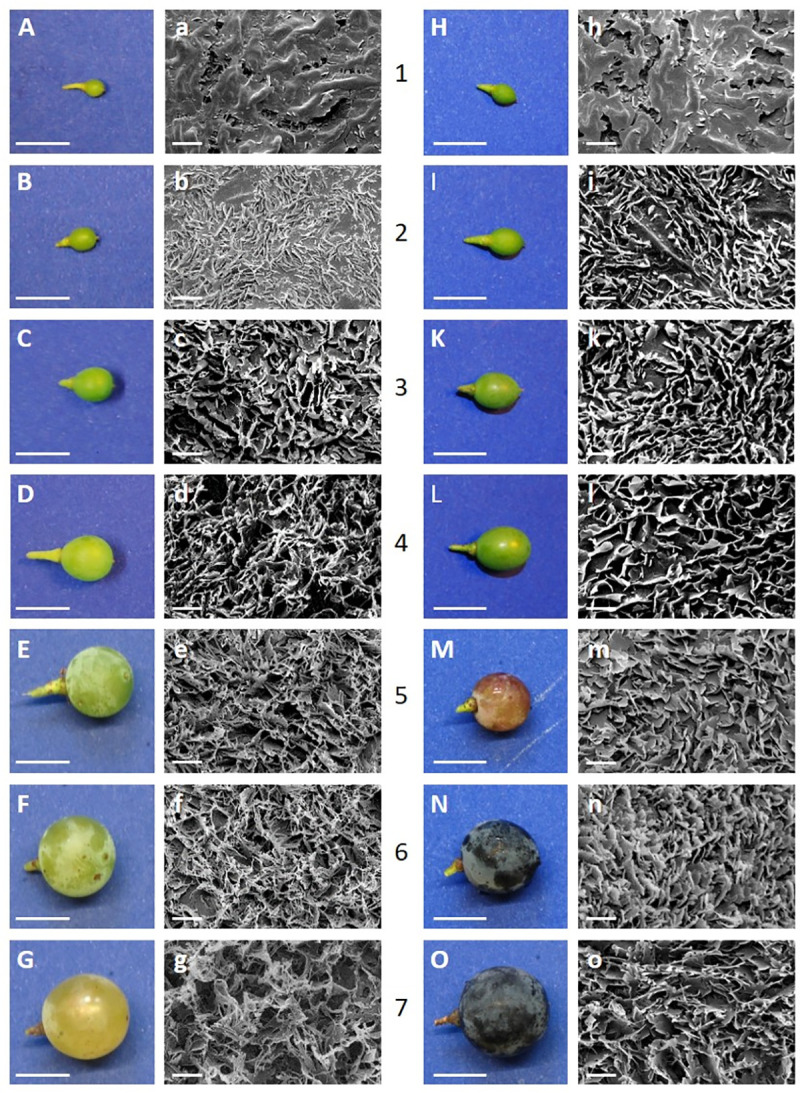
Morphology of grape berries (uppercase) and the respective cryo-scanning electron micrographs (8.000x) of the epicuticular wax crystals (lowercase) of *V*. *vinifera* cv. Müller Thurgau (left) and cv. Blauer Spätburgunder (right) during development (category 1–7).

Grape berry development of both cultivars MTH and BSB was almost simultaneous and followed a bi-sigmoidal growth pattern with a rapid increase in size during the first two weeks after berry set (Fig 1) followed by a slightly pronounced lag phase with less growth in July (Fig 1). Upon veraison in the beginning of august, growth resumed, sudden change in color became visible and berries softened (Fig 1E–1G and 1M–1O).

**Fig 2:**
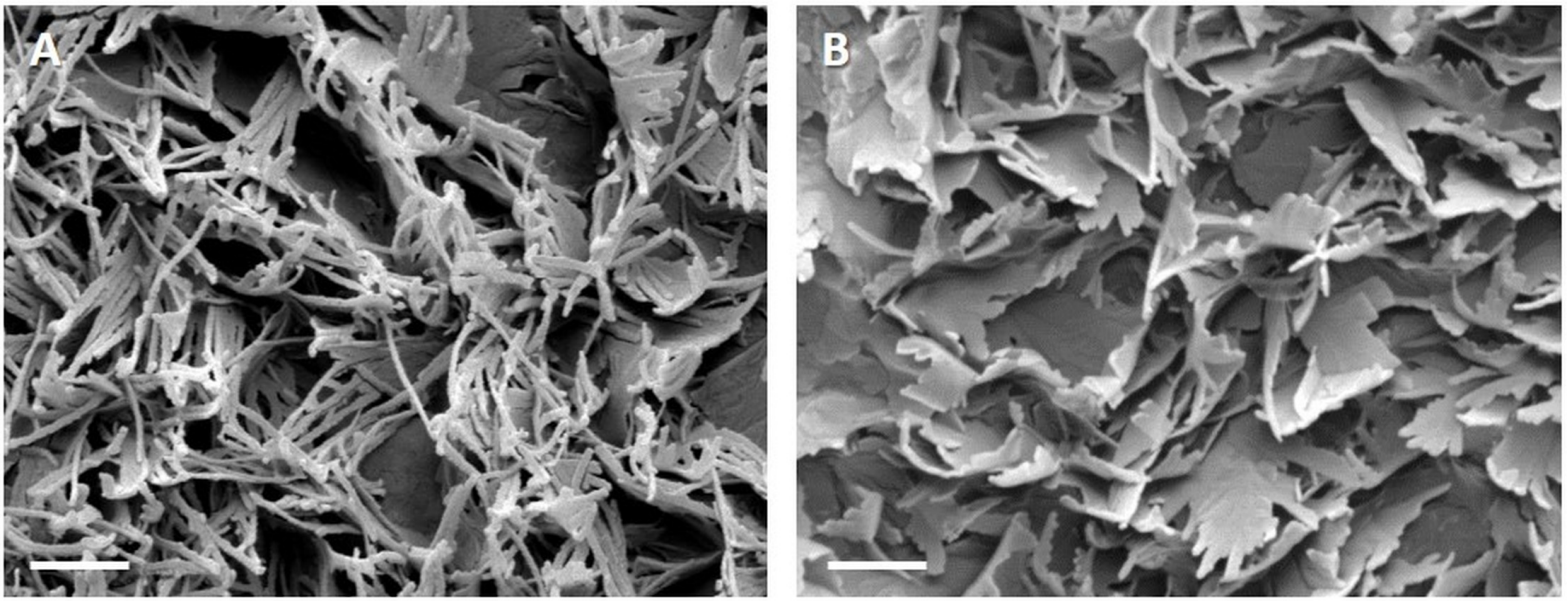
Surface of a berry in full maturity with epicuticular wax crystals recorded with cryo-scanning electron microscope (8.000x). A, In the cultivar Müller-Thurgau epicuticular wax appears as membranous platelets with distinct threadlike extensions. B, Cultivar Blauer Spätburgunder (Pinot noir) shows platelets with rounded margins and on the upper side with crenated edges.

In both cultivars, epicuticular wax crystals arised early in the fruit development as a gentle glaucous coating, covering the entire fruit surface (Fig 1A–1D, 1H–1L). When berries began to touch each other (BBCH 77), the wax coat appeared patchier and during maturation, glossy spots became visible (Fig 1E–1G, 1M–1O). The detailed morphological pattern of epicuticular wax crystal formation during fruit development was investigated with Cryo-SEM. At fruit set, were fruits began to swell (BBCH 71), cuticular ridges started to spread and the surface in between was covered with a fractured crust according to Barthlott *et al*. (1998) [27]. Underneath the fissures, some primary wax crystals became visible in both cultivars (Fig 1A–1H). One week later (category 2), non-entire parallel stacked platelets protruded perpendicularly from the underlying wax film (Fig 1B–1I). At some sites above ridges, the underlying cuticle or wax film was still visible. During further development, the crystals grew to a dense layer completely covering the cuticle beneath. The basic structure of the wax crystals of mature berries of both varieties studied consists of platelets according to Barthlott et al. (1998) [27] that protrude from the surface at a more or less acute angle. The orientation of the platelets of different heights appears random, resulting in a very irregular pattern. In MTH epicuticular wax appears as membranous platelets with threadlike extensions (Figs 2A and 1C–1G), which became more distinct during ripening (BBCH 81–89). In BSB the “young” non-entire platelets had rounded margins which became more and more crenated until fruit maturity (Figs 2B and 1K–1O).

### The pattern of total cuticular wax during berry development is highly dynamic

The wax of grape berries of the cultivars MTH and BSB consisted of two fractions (S2 and S3 Figs). The cyclic fraction dominated the entire grape berry wax. Beside the major triterpenoid oleanolic acid also minor amounts of its aldehyde and methyl ester derivatives, erythrodiol, other triterpenoids and several steroids and tocopherols were detected (S2 and S3 Figs). The very-long-chain aliphatics (VLCA) consisted of alcohols (C22-C32), fatty acids (C20-C34), aldehydes (C22-C32) and alkyl esters (C38-C58) predominantly with even chain numbers (S2 and S3 Figs). Alkanes were only found in traces and are therefore not further considered. Beside others, the non-identified fraction included two homologous serious with single diagnostic ions at 280 m/z and 369 m/z and molecular masses in the range of C36-C44 alkyl esters.

**Fig 3:**
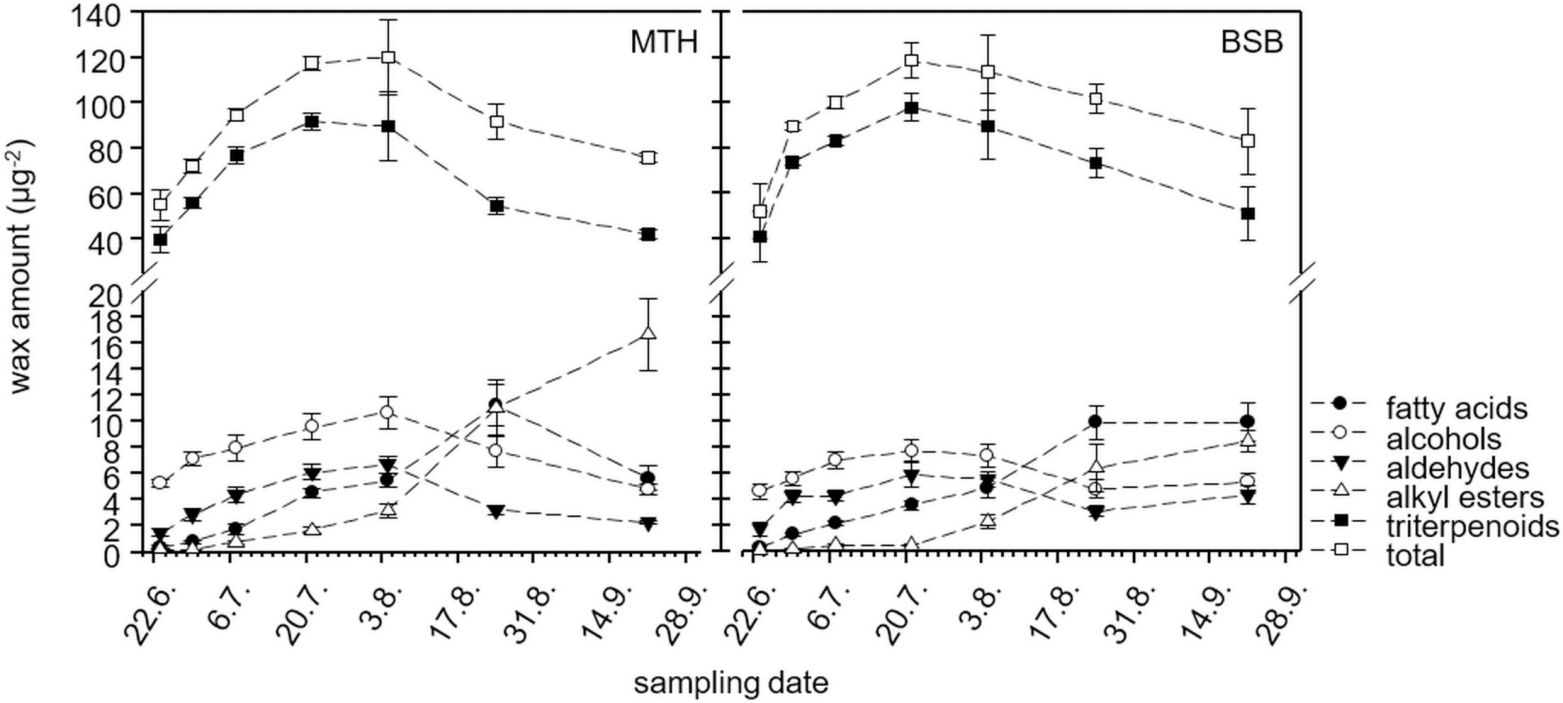
Developmental pattern of different substance classes within the total wax (wax crystals plus remaining wax) of *V*. *vinifera* cv. Müller Thurgau (left) and cv. Blauer Spätburgunder (right) berries (N = 5, mean ± SD).

The amount and composition of the total cuticular wax was highly dynamic during development. In very young berries (category 1) about 40 μg cm^-2^ triterpenoids were present in the cuticle of both cultivars (Fig 3). The triterpenoid coverage increased significantly to 91.9 ± 3.9 μg cm^-2^ in MTH and 98.0 ± 6.1 μg cm^-2^ in BSB until veraison. From the beginning of august where ripening proceeds (category 5), the triterpenoid coverage per fruit surface area decreased again. At maturation, it reached comparable values as in the beginning of the fruit development (42.3 ± 1.7 μg cm^-2^ in MTH and 51.4 ± 11.7 μg cm^-2^ in BSB, Fig 3), where aliphatics represented about 15% of the total wax (S4 Fig). The total amount of aliphatics increased in a similar rate than the triterpenoids, keeping the proportion nearly constant until veraison (S4 Fig). With the beginning of fruit softening (category 5), where triterpenoid coverage decreased, the total aliphatic coverage increased further reaching a proportion of 38.4 ± 5.5% of the total wax in MTH and 35.9 ± 3.4% in BSB (S4 Fig) at maturity. However, different substance classes of the aliphatics behave different during berry development (Fig 3). In both cultivars the amount of fatty acids, alcohols and aldehydes per surface area increased from the very early fruit development and stagnated or rather decreased again during berry ripening, where a strong increase in alkyl esters was observed (Fig 3). This effect was much more pronounced in MTH where alkyl esters resulted in a final coverage of 16.6 ± 2.7 μg cm^-2^, which was almost twice as much as in BSB (8.5 ± 0.8 μg cm^-2^) (S5 Fig). Simultaneously, the quantity of fatty acids and aldehydes at maturity was significantly reduced in MTH compared to BSB (S5 Fig). Detailed information about statistical differences is given in S5 Fig.

### Epicuticular wax crystals are almost exclusively composed of long chain aliphatics

**Fig 4:**
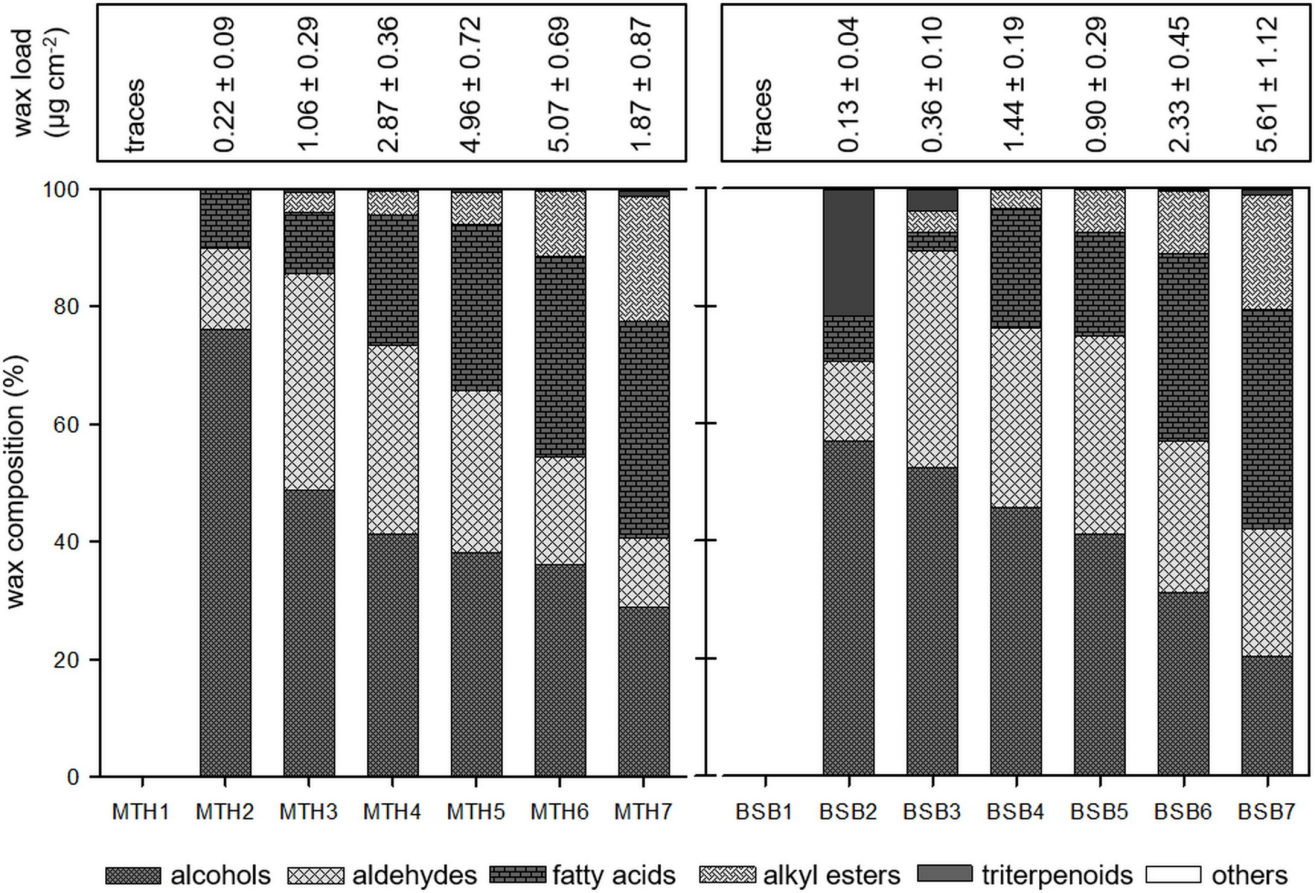
Amount of epicuticular wax crystals (μg cm^-^^2^) and compositional changes concerning different substance classes during berry development of *V*. *vinifera* cv. Müller Thurgau (left) and cv. Blauer Spätburgunder (right) (N = 5, mean ± SD).

**Fig 5:**
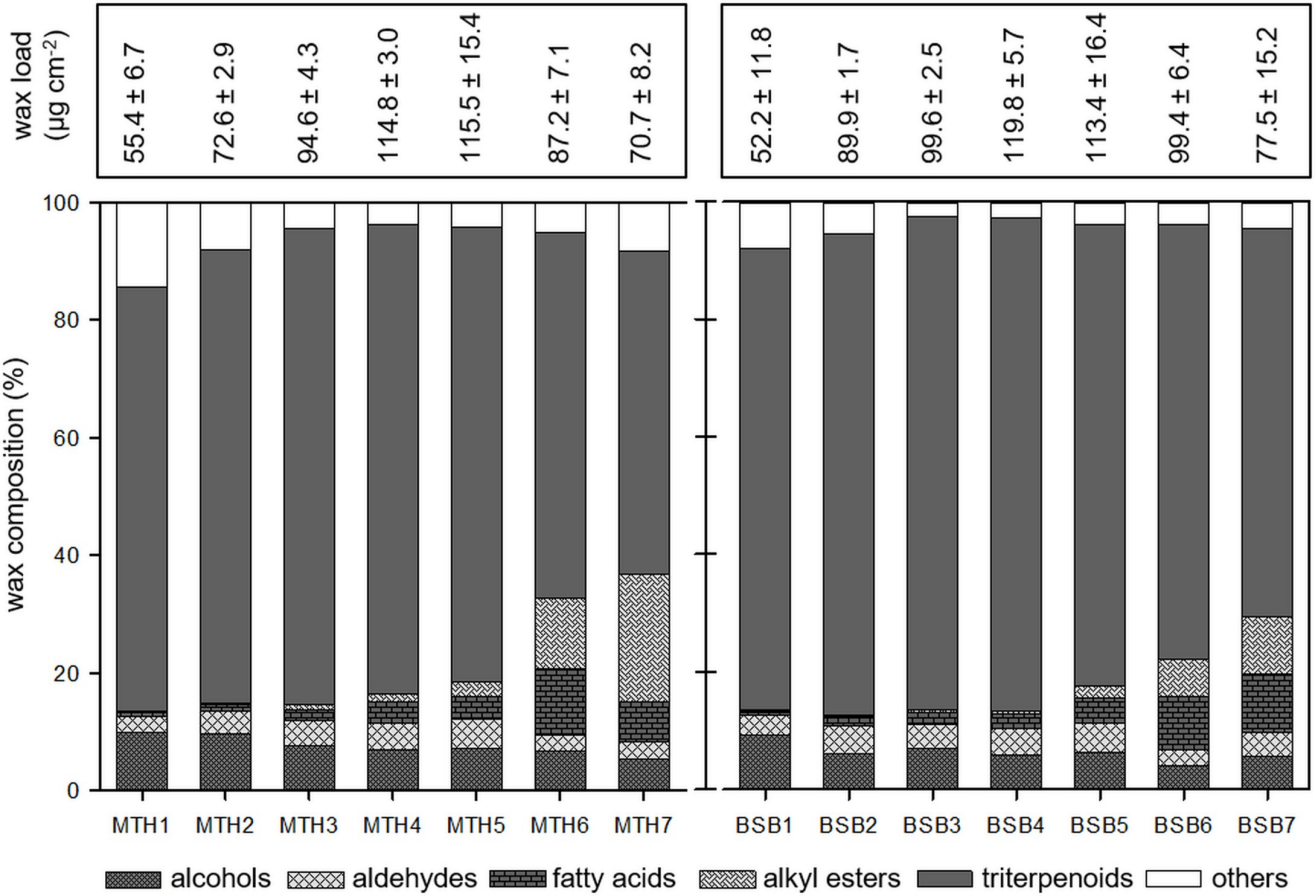
Amount (μg cm^-^^2^) of remaining wax after mechanical removal of the wax crystals and compositional changes concerning different substance classes during berry development of *V*. *vinifera* cv. Müller Thurgau (left) and cv. Blauer Spätburgunder (right) (N = 5, mean ± SD).

The composition of epicuticular wax crystals and underlying wax was investigated separately by mechanical removal of the outer wax crystals from the surface of grape berries followed by chemical extraction of the remaining wax. The crystals amounted only to a small portion compared to the remaining wax fraction (Figs 4 and 5). They were solely composed of very long chain aliphatics while triterpenoids were almost completely missing (Fig 4). In very young berries (category 1) only traces of typical wax components were found. From category 2, the load of epicuticular wax crystals increased in both cultivars and was always higher in MTH compared to BSB except for the last stage where it decreased again in MTH (Fig 4). In the beginning of fruit development epicuticular wax crystals were dominated by alcohols (about 76% in MTH, 57% in BSB) and smaller proportions of aldehydes and fatty acids were present. The proportions of alcohols and aldehydes decreased during berry formation and ripening in both cultivars while the proportion of fatty acids increased. At BBCH 75 (category 3) alkyl esters appeared and the proportion increased to about 20% of the total wax crystal composition at maturity in both cultivars (category 7).

### The remaining wax coverage is dominted by cyclic triterpenoids

The remaining wax coverage per surface area was much higher than the epicuticular wax crystals. It was dominated by cyclic triterpenoids (Fig 5) with oleanolic acid representing the main portion of about 85% (MTH1) to 95% (MTH4 and BSB3), depending on the developmental stage. In younger fruits, the portion of aliphatics was about 20%. It increased from veraison (category 5) were alkyl esters appeared in significant amounts (Fig 5). Upon maturity, alkyl esters comprised 9.8 ± 1.8% of the total intracuticular wax in BSB and 21.9 ± 3.5% in MTH.

## Discussion

The overall composition of grape berry cuticular wax has been studies for more than five decades [4, 8, 26]. However, only one report compared the chemical plasticity and morphology of the entire cuticular wax (including epi- and intracuticular wax) during grape berry development [28] by using TLC analysis, that allowed only for a crude separation. Albeit the relative wax composition varies between cultivars, oleanolic acid was reported to be the dominant compound in all investigated grape berry waxes [7, 8, 29, 30]. We also identified oleanolic acid to be the main compound in the total cuticular fruit wax of both investigated *V*. *vinifera* cultivars, ‘Müller Thurgau’ and ‘Blauer Spätburgunder’. Furthermore, we found remarkable differences were found between the composition of the epicuticular wax bloom and the underlying wax which is partially associated with the cutin matrix. The epiculticular wax on the surface represent the interface between the berry and the environment. Therefore, the chemical and structural properties of this boundary layer are highly important for coping with abiotic and biotic external influences such as UV-radiation, dehydration, pests and pathogens.

The epicuticular wax crystals, appear early in the fruit development as a glaucous covering on the surface (Fig 1). They totally lack of triterpenoids and are exclusively composed of very-long-chain aliphatic (VLCA) compounds (Fig 4). This pattern was already shown by Possingham et al. (1967) [5] for ripe grape berries of the variety sultana. They found VLCA alcohols to be the main compound beside smaller amounts of acids, esters, aldehydes and hydrocarbons. However, by looking at ripe fruits only a snapshot of the wax composition is mirrored.

The wax chemistry is adapted to the vigorous growth of the berryIndeed, not only the wax amount but also the composition was shown to be highly dynamic during development. The amount of VLCA fatty acids significantly increases during the fruit development and VLCA alkyl esters occur and increase only later during development (from BBCH 75 on). At any time, triterpenoids are solely located underneath the wax crystals together with additional VLCAs. Khanal et al. (2013) [31] supposed that the stresses and strains that occur during the fruit development as the surface expands are fixed by the current deposition of wax in the cutin, thereby converting reversible elastic into irreversible plastic strain. Additionally, triterpenoids, were shown to be of significant importance for maintaining the mechanical strength of the cuticle [14].

During the later stages, when berry expansion declines, the amount of triterpenoids decrease, as was also previously shown for cherry fruits [32] while the proportion of VLCAs significantly increases (Fig 3 and S4 Fig). From then, substantial amounts of VLCA fatty acids and alkyl esters are deposited in the cuticular wax, leading to significant increase in the proportion of VLCAs up to about 35–40% of the total wax load. VLCA wax compounds rather than triterpenoids are responsible for the permeation barrier in the cuticle [13, 33]. During development, triterpenoids are completely shielded against the environment by overlaying VLCAs which also build a dense bloom of wax crystals on the surface (Fig 1). This became obvious during the experimental procedure. In the beginning, triterpenoids could be extracted by immersing the intact berries in methanol due to their higher polarity compared to VLCAs (personal observation). At developmental stage 3 (BBCH 75) no triterpenoids could be detected any more in methanol extracts of intact fruits since the overlaying VLCAs prevent the methanol from entering the cuticle to extract them. Contrary, in chloroform extracts, both VLCAs and triterpenoids are found. The chloroform dissolves the VLCAs shielding the outer surface of the cuticle, granting access to the intracuticular triterpenoids. Although, remarkable differences in epi- and intracuticular wax was already shown for leaves [13, 34] and fruits [5, 35] of several plant species, dissolving fruits or leaves in chloroform is still a very common method to obtain wax from plant surfaces. With this method, the extracted wax always represents a mixture of epi- and intracuticular wax with variable proportions, depending on the extraction time. This should be considered when the function of wax is related to its chemical composition because the intracuticular wax do not face the environment.

The wax chemistry effects the crystal architecture The cutin network which is already formed before anthesis [36] might play an important role as stationary phase interacting more or less with the wax molecules, depending on their dimension or polarity. It was proposed that the asymmetric cutin matrix has an open branching/network structure with the presence of cavities, which may potentially be occupied by wax molecules [37]. Due to the relatively high polarity of the triterpenoids compared to VLCAs, one would expect that they are captured in the inner layer of the cuticle which is more polar due to the interaction with cell wall polysaccharides. Wide-angle X-ray diffraction and NMR analysis of persimmon fruit cuticles showed that triterpenoids construct a nanocomposite in the cuticular matrix, while VLCAs did not exhibit clear interactions with cutin [14]. They might be transported further via the transpiration stream or simply move by diffusion along a solubility/polarity gradient, finally accumulating as wax film on the surface. Furthermore, the self-assembly process of wax molecules leads to the growth of three-dimensional crystalline structures emerging from the underlying wax film [38]. Thereby, the wax chemistry has a formative influence [39]. In most plant species, the different architectures of the crystals are associated with a single dominating constituent or compound class. While tubules are crystallized from secondary alcohols or ß-diketones, platelets are dominated by primary alcohols, aldehydes or ketones of a certain chain length. In the case of heterogeneous wax mixtures, usually glossy wax films are observed [39, 40]. However, in grape berries distinct crystal architecture is observed by SEM despite the fact that we found a heterogeneous mixture of numerous compound classes and chain length. Nevertheless, at berry set, were alcohols clearly dominate the epicuticular wax (Fig 5) the typical platelets are observed (Fig 1). During berry development, the wax mixture becomes progressiveley heterogeneous which also affect the crystal structure. With increasing chemical heterogeneity, the initial order yield to an irregular pattern of crenated and fissured wax lobes (Fig 2). In ripe berries, the chemical differences in the epicuticular wax of both cultivars with higher proportions of aldehydes and less alcohols in BSB (Fig 5) is also mirrored by the architecture of the wax crystals. In MTH a separation of the lobes into filamentous structures was strongly pronounced (Fig 2).

### Aspects of wax crystal formation in fruit defense against pathogens

The chemical composition of VLCAs combined with the specific three-dimensional structure resulting from the self-assembly of the wax compounds, leads to a rough super-hydrophobic surface which is very hard-to-wet [41]. Water is repelled very easy and during run off, water droplets will clean the surface from dust and other particles including fungal spores or bacteria [40, 42, 43], which do not adhere actively to the waxy surface. Due to its specific architecture at the beginning of development, the berry is adapted to the exponential increase in berry volume and thereby the rapid expansion of the berry surface at this stage. In this way epicuticular wax crystals arise very early in the fruit development shielding the underlying cuticle and intracuticular waxes against pathogen attack such as infections by powdery and downy mildew [18] within two to three weeks after bloom (BBCH 75). It was shown, that the resistance of diverse grape cultivars to the infestation with *Botrytis cinerea* increases with increasing amount of cuticular wax, while the susceptibility of the ripe berries drastically increased at contact sites between berries, where the epicuticular wax was altered [19, 20]. So far it remains unclear, whether the infection success of several pathogens is initiated or suppressed by the presence or absence of specific cuticle constituents at different stages of grape berry development. By studying berry-pathogen or berry-insect interactions and mite attack, fundamental differences in the chemical composition of epicuticular wax crystals and the underlying wax have to be considered in the future, since wax crystals completely lacking of triterpenoids are the first contact site between grape berries and pests.

## Supporting information

S1 Fig Comparison of chromatographs (GC-FID) for different extraction methods of epicuticular waxes of *Vitis vinifera* cv. Müller Thurgau berries (category 3). 1: deionized water with 5 min ultra-sonic, 2: 10% ethanol in saturated aqueous sodium chloride solution without ultra-sonic, 3: 10% ethanol in saturated aqueous sodium chloride solution with 5 min ultra-sonic. IS: internal standard n-tetracosane (3 μg).
(TIF)

S2 Fig Comparison of chromatographs (GC-FID) for different extraction methods of epicuticular wax crystals (mechanical removal) and the underlying wax (MeOH and CHCl_3_ extraction) of *Vitis vinifera* cv. Müller Thurgau berries (category 3). IS: internal standard n-tetracosane added in different amounts (mechanical removal: 5 μg, MeOH extraction: 25 μg, CHCl_3_ extraction: 10 μg).
(TIF)

S3 Fig Detailed composition of the total wax (epi- plus intracuticular) of three representative developmental stages (1, 4, 7) of *Vitis vinifera* cv. Müller Thurgau berries. The numbers on the x-Axis refer to the carbon chain length of the aliphatic compounds. (N = 5, Mean + SD).
(TIF)

S4 Fig Detailed composition of the total wax (epi- plus intracuticular) of three representative developmental stages (1, 4, 7) of *Vitis vinifera* cv. Blauer Spätburgunder berries. The numbers on the x-Axis refer to the carbon chain length of the aliphatic compounds. (N = 5, Mean + SD).
(TIF)

S5 Fig Proportional change of cyclic and aliphatic fractions in the total cuticular wax of *Vitis vinifera* cv. Müller Thurgau (MTH) and cv. Blauer Spätburgunder (BSB) berries during fruit development. (N = 5, Mean ± SD).
(TIF)

S6 Fig Developmental changes in the coverage (μg cm^-2^) of individual substance classes within the total wax of *Vitis vinifera* cv. Müller Thurgau (MTH) and cv. Blauer Spätburgunder (BSB) berries. Two-way analysis of variance was performed within substance classes and all pairwise multiple comparison procedure (Holm-Sidak method) was used to detect significant differences (indicated by different letters) within cultivars during development (MTH: uppercase, BSB: lowercase) and between both cultivars at a given developmental stage (*: p < 0.05, **: p < 0.001, DF = 66, Mean + SD).
(TIF)
