# Supplementary figures and images for: Developmental pattern of grapevine (Vitis vinifera L.) berry cuticular wax: Differentiation between epicuticular crystals and underlying wax
Source: PLoS One. 2021 Feb 19;16(2):e0246693. doi: 10.1371/journal.pone.0246693 (PMC7894928; doi:10.1371/journal.pone.0246693)

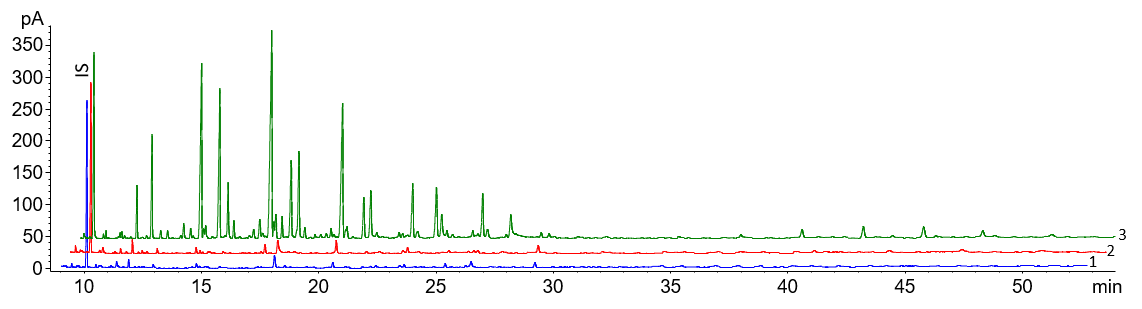

Supplement: S1 Fig — 1: deionized water with 5 min ultra-sonic, 2: 10% ethanol in saturated aqueous sodium chloride solution without ultra-sonic, 3: 10% ethanol in saturated aqueous sodium chloride solution with 5 min ultra-sonic. IS: internal standard n-tetracosane (3 μg). (TIF) [file pone.0246693.s001.tif]

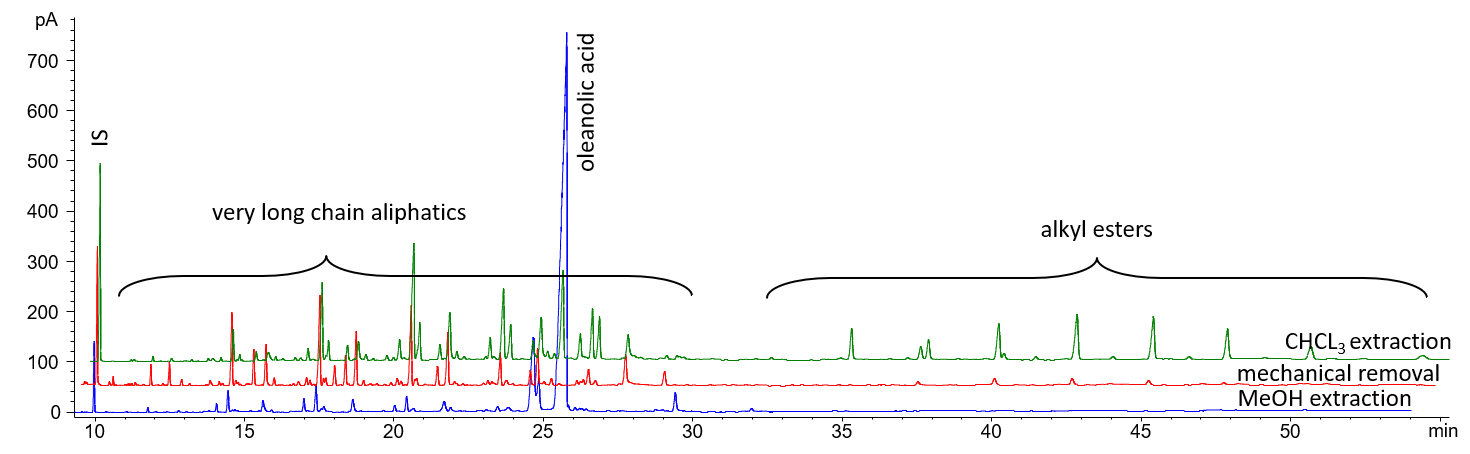

Supplement: S2 Fig — IS: internal standard n-tetracosane added in different amounts (mechanical removal: 5 μg, MeOH extraction: 25 μg, CHCl3 extraction: 10 μg). (TIF) [file pone.0246693.s002.tif]

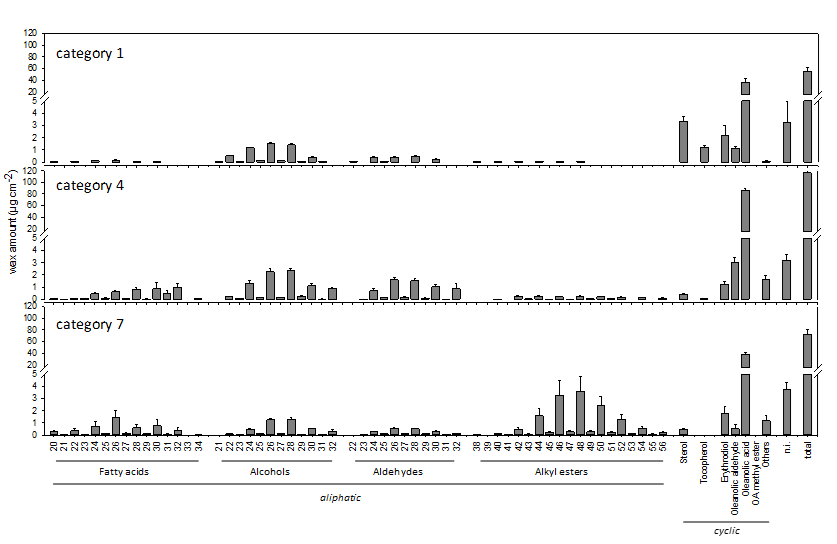

Supplement: S3 Fig — The numbers on the x-Axis refer to the carbon chain length of the aliphatic compounds. (N = 5, Mean + SD). (TIF) [file pone.0246693.s003.tif]

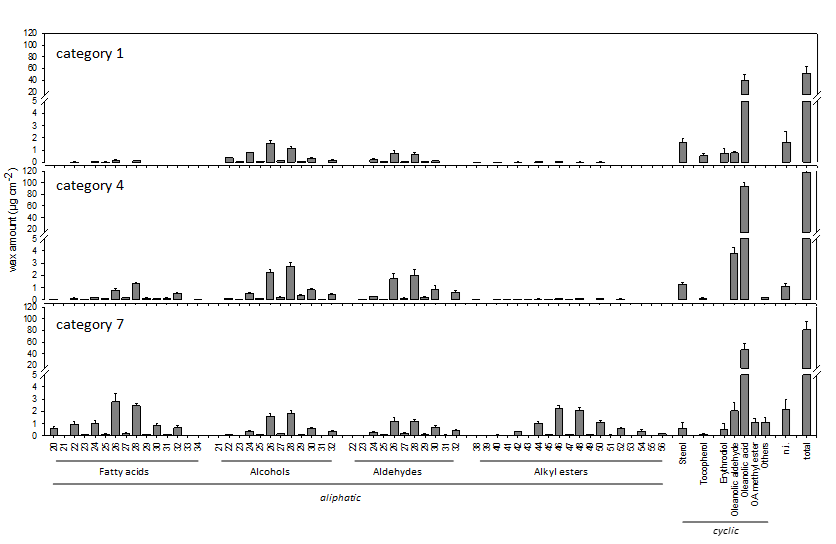

Supplement: S4 Fig — The numbers on the x-Axis refer to the carbon chain length of the aliphatic compounds. (N = 5, Mean + SD). (TIF) [file pone.0246693.s004.tif]

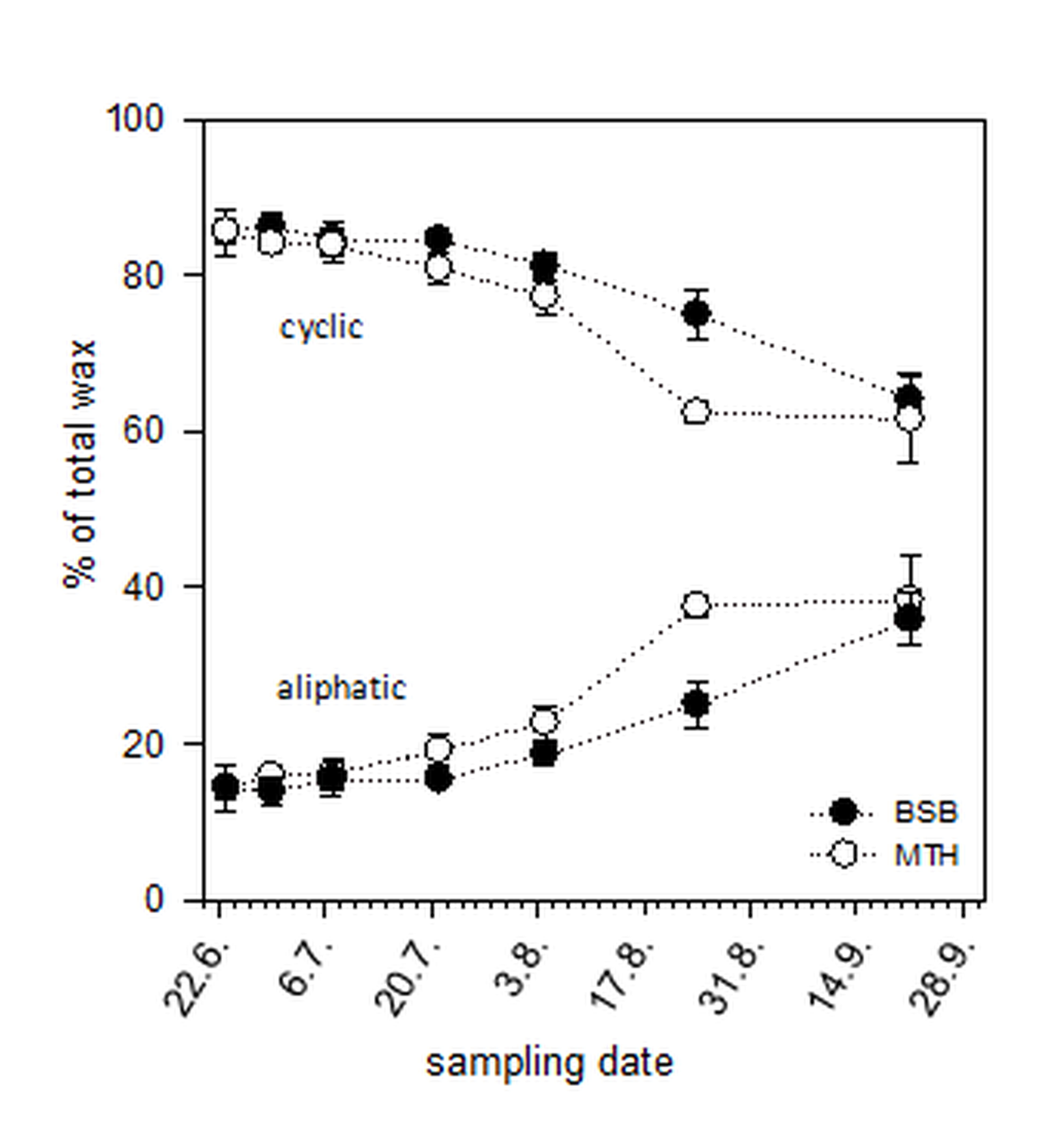

Supplement: S5 Fig — (N = 5, Mean ± SD). (TIF) [file pone.0246693.s005.tif]

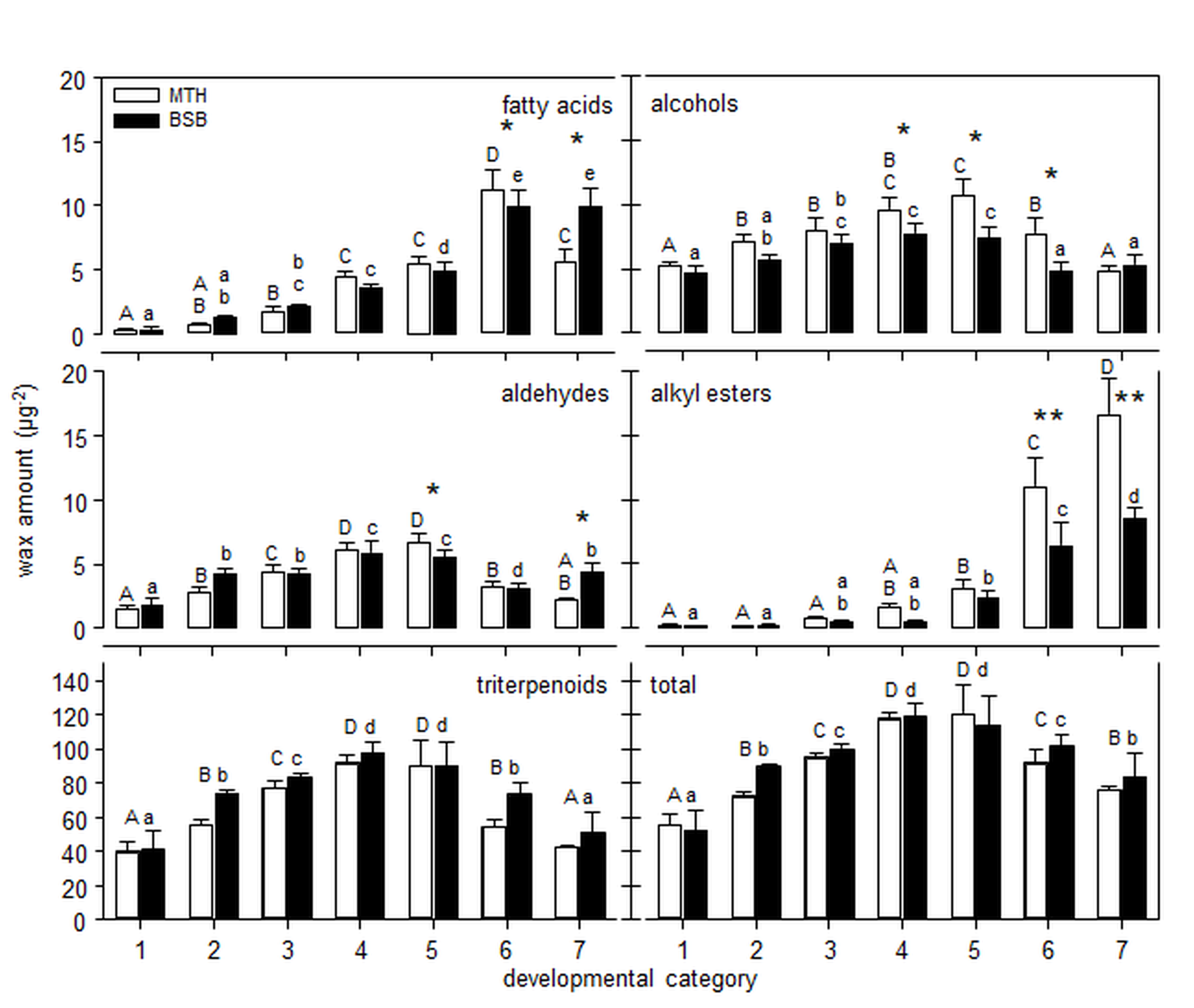

Supplement: S6 Fig — Two-way analysis of variance was performed within substance classes and all pairwise multiple comparison procedure (Holm-Sidak method) was used to detect significant differences (indicated by different letters) within cultivars during development (MTH: uppercase, BSB: lowercase) and between both cultivars at a given developmental stage (*: p < 0.05, **: p < 0.001, DF = 66, Mean + SD). (TIF) [file pone.0246693.s006.tif]
