# Supplementary material for: Developmental pattern of grapevine (Vitis vinifera L.) berry cuticular wax: Differentiation between epicuticular crystals and underlying wax
Source: PLoS One. 2021 Feb 19;16(2):e0246693. doi: 10.1371/journal.pone.0246693 (PMC7894928; doi:10.1371/journal.pone.0246693)
Supplement: Attachment — Submitted filename: PONE-D-20-32426.HG.pdf [file pone.0246693.s007.pdf]

PLOS ONE

Developmental pattern of grapevine (Vitis vinifera L.) berry cuticular wax: differentiation between epicuticular crystals and underlying wax

--Manuscript Draft--

**Manuscript Number:**	PONE-D-20-32426
**Article Type:**	Research Article
**Full Title:**	Developmental pattern of grapevine (Vitis vinifera L.) berry cuticular wax: differentiation between epicuticular crystals and underlying wax
**Short Title:**	Grapevine berry cuticula and epicuticular crystals
**Corresponding Author:**	Hanns-Heinz Kassemeyer<br>Staatliches Weinbauinstitut<br>Freiburg, GERMANY
**Keywords:**	Berry skin, fruit cuticle, grapevine, epicuticular wax crystals, triterpenoids, very-long-chain aliphatics, Vitis vinifera
**Abstract:**	The grapevine berry surface is covered by a cuticle consisting of cutin and various lipophilic wax compounds. The latter build the main barrier for transpirational water loss and protect the fruit against environmental factors e.g. pests, mechanical impacts or radiation. The integrity of the fruit surface is one important key factor for post-harvest quality and storage of fruits. Nonetheless, the developmental pattern of cuticular wax was so far only investigated for a very limited number of fruits. Therefore, we performed comparative investigations on the compositional and morphological nature of epicuticular wax crystals and underlying wax during fruit development in Vitis vinifera . The main compound oleanolic acid belongs to the pentacyclic triterpenoids, which occur very early in the development in high amounts inside the cuticle. The amount increases until veraison and decreases further during ripening. In general, very-long chain aliphatic (VLCA) compounds are present in much smaller amounts but alcohols and aldehydes follow the same trend during development. In contrast, the amount of fatty acids constantly increases from fruit set to ripening while wax esters only occur in significant amount at veraison and increase further. Wax crystals at the fruit surface are solely composed of VLCAs and the morphology changes during development according to the compositional changes of the VLCA wax compounds. The remarkable compositional differences between epicuticular wax crystals and the underlying wax are important to understand in terms of studying grape-pest interactions or the influence of environmental factors, since only wax crystals directly face the environment.
**Order of Authors:**	Hanns-Heinz Kassemeyer
	Katja Arand
	Evi Bieler
	Markus Dürrenberger
**Opposed Reviewers:**	Antonio Heredia<br>Instituto de Hortofruticultura Subtropical y Mediterránea (ISM)-La Mayora, Departamento de Mejora Genética y Biotecnología (CSIC), Algarrobo-Costa, E-29750 Málaga,<br><br>There is a collaboration with one of the authors
	Gloria Gloria López Casado<br>Universidad de Málaga: Universidad de Malaga<br><br>There is a collaboration with one of the authors
**Additional Information:**	
**Question**	**Response**
**Financial Disclosure**	This work was partly supported by the European Union program to support cross-

Powered by Editorial Manager® and ProduXion Manager® from Aries Systems Corporation

Enter a financial disclosure statement that describes the sources of funding for the work included in this submission. Review the submission guidelines for detailed requirements. View published research articles from *PLOS ONE* for specific examples.<br><br>This statement is required for submission and **will appear in the published article** if the submission is accepted. Please make sure it is accurate.<br><br>**Unfunded studies**<br>Enter: *The author(s) received no specific funding for this work.*<br><br>**Funded studies**<br>Enter a statement with the following details:<br>• Initials of the authors who received each award<br>• Grant numbers awarded to each author<br>• The full name of each funder<br>• URL of each funder website<br>• Did the sponsors or funders play any role in the study design, data collection and analysis, decision to publish, or preparation of the manuscript?<br>• **NO** - Include this sentence at the end of your statement: *The funders had no role in study design, data collection and analysis, decision to publish, or preparation of the manuscript.*<br>• **YES** - Specify the role(s) played.<br><br>* typeset	border cooperation INTERREG V Upper Rhine 2014-2020 (grant no. 1607) VITIFUTUR - Transnational Platform for Applied Research and Further Training in Viticulture.
**Competing Interests**<br><br>Use the instructions below to enter a competing interest statement for this submission. On behalf of all authors, disclose any competing interests that could be perceived to bias this work—acknowledging all financial support and any other relevant financial or non-financial competing interests.<br><br>This statement **will appear in the published article** if the submission is accepted. Please make sure it is accurate. View published research articles	The authors have declared that no competing interests exist.

from *PLOS ONE* for specific examples.<br><br>**NO authors have competing interests**<br><br>Enter: *The authors have declared that no competing interests exist.*<br><br>**Authors with competing interests**<br><br>Enter competing interest details beginning with this statement:<br><br>*I have read the journal's policy and the authors of this manuscript have the following competing interests: [insert competing interests here]*<br><br>* typeset	
**Ethics Statement**<br><br>Enter an ethics statement for this submission. This statement is required if the study involved:<br><br>• Human participants<br>• Human specimens or tissue<br>• Vertebrate animals or cephalopods<br>• Vertebrate embryos or tissues<br>• Field research<br><br>Write "N/A" if the submission does not require an ethics statement.<br><br>General guidance is provided below. Consult the submission guidelines for detailed instructions. **Make sure that all information entered here is included in the Methods section of the manuscript.**	N/A

**Format for specific study types**<br><br>**Human Subject Research (involving human participants and/or tissue)**<br>• Give the name of the institutional review board or ethics committee that approved the study<br>• Include the approval number and/or a statement indicating approval of this research<br>• Indicate the form of consent obtained (written/oral) or the reason that consent was not obtained (e.g. the data were analyzed anonymously)<br><br>**Animal Research (involving vertebrate animals, embryos or tissues)**<br>• Provide the name of the Institutional Animal Care and Use Committee (IACUC) or other relevant ethics board that reviewed the study protocol, and indicate whether they approved this research or granted a formal waiver of ethical approval<br>• Include an approval number if one was obtained<br>• If the study involved *non-human primates*, add *additional details* about animal welfare and steps taken to ameliorate suffering<br>• If anesthesia, euthanasia, or any kind of animal sacrifice is part of the study, include briefly which substances and/or methods were applied<br><br>**Field Research**<br><br>Include the following details if this study involves the collection of plant, animal, or other materials from a natural setting:<br>• Field permit number<br>• Name of the institution or relevant body that granted permission	
**Data Availability**<br><br>Authors are required to make all data underlying the findings described fully available, without restriction, and from the time of publication. PLOS allows rare exceptions to address legal and ethical concerns. See the PLOS Data Policy and FAQ for detailed information.	Yes - all data are fully available without restriction

Powered by Editorial Manager® and ProduXion Manager® from Aries Systems Corporation

A Data Availability Statement describing where the data can be found is required at submission. Your answers to this question constitute the Data Availability Statement and **will be published in the article**, if accepted.<br><br>**Important:** Stating 'data available on request from the author' is not sufficient. If your data are only available upon request, select 'No' for the first question and explain your exceptional situation in the text box.<br><br>Do the authors confirm that all data underlying the findings described in their manuscript are fully available without restriction?	
**Describe where the data may be found in full sentences. If you are copying our sample text, replace any instances of XXX with the appropriate details.**<br><br>• If the data are **held or will be held in a public repository**, include URLs, accession numbers or DOIs. If this information will only be available after acceptance, indicate this by ticking the box below. For example: *All XXX files are available from the XXX database (accession number(s) XXX, XXX.*).<br>• If the data are all contained **within the manuscript and/or Supporting Information files**, enter the following: *All relevant data are within the manuscript and its Supporting Information files.*<br>• If neither of these applies but you are able to provide **details of access elsewhere**, with or without limitations, please do so. For example:<br><br>*Data cannot be shared publicly because of [XXX]. Data are available from the XXX Institutional Data Access / Ethics Committee (contact via XXX) for researchers who meet the criteria for access to confidential data.*<br><br>*The data underlying the results presented in the study are available from (include the name of the third party*	All relevant data are within the manuscript and its Supporting Information files.

Powered by Editorial Manager® and ProduXion Manager® from Aries Systems Corporation

and contact information or URL).<br>• This text is appropriate if the data are owned by a third party and authors do not have permission to share the data.<br><br>* typeset	
Additional data availability information:	Tick here if your circumstances are not covered by the questions above and you need the journal's help to make your data available.

Powered by Editorial Manager® and ProduXion Manager® from Aries Systems Corporation

Prof. Dr. Hanns-Heinz Kassemeyer
State Institute for Viticulture Freiburg
Merzhauser Str. 119
79100 Freiburg Germany
hanns-heinz.kassemeyer@wbi.bwl.de

Dear Editor,

Please find attached our research article entitled ‘Developmental pattern of grapevine (*Vitis vinifera* L.) berry cuticular wax: differentiation between epicuticular crystals and underlying wax’ prepared by K. Arand *et al*. Due to our submission to PLOS BIOLOGY, we were invited to submit the current manuscript to PLOS ONE.

In our work we have analyzed chemical composition and nanostructure of cuticular waxes of grapevine berries during development from berry setting to maturation. With the special method of Cryo-Scanning-Electron-Microscopy (Cryo-SEM) we preserved the *in-situ* state of the epicuticular waxes without denaturing fixation steps. In combination with the selective extraction and analysis of the epicuticular wax crystals and the remaining wax, we gained new insights into the surface development of berries on both chemical and structural level. We build our experiments on prior grapevine publications, showing (i) heterogeneous wax composition between epi- and intracuticular wax only in ripe berries based on considerably basic experimental procedures (TLC), (ii) the ultrastructure of cuticular wax during development without chemical composition and (iii) developmental changes in triterpenoid content of cuticular waxes leaving the aliphatic wax fraction out of consideration.

Knowledge of the surface structure and chemistry of fruits is gaining scientific and applied importance for a variety of reasons. (i) They play a significant role in coping with abiotic stress especially in terms of climate changes. (ii) They are a preformed barrier in the interaction with pathogens and potentially act as trigger for the formation of infectious structures. (iii) Especially in fruits, they play an important role in postharvest handling and quality. (iv) Last but not least, they are of great interest for bionics and research on bioinspired materials.

The present manuscript has not been published and is not under consideration for publication, elsewhere. All persons entitled to authorship have been named, and each approved the final version of the submitted manuscript.

Yours sincerely,

Hanns-Heinz Kassemeyer

Manuscript
Click here to access/download;Manuscript;Arand_et_al_Okt. 2020_KA manuscript.docx

Developmental pattern of grapevine (*Vitis vinifera* L.) berry cuticular wax: differentiation between epicuticular crystals and underlying wax

Katja Arand[1], Evi Bieler[2], Markus Dürrenberger[2], Hanns-Heinz Kassemeyer[3,4]*

[1]University of Würzburg, Julius von Sachs Institute for Biosciences, Würzburg, Germany

[2]University of Basel, Swiss Nanoscience Institute - Nano Imaging Lab, Basel, Switzerland

[3]State Institute for Viticulture, Freiburg, Germany

[4]University of Freiburg, Institute of Biology II, Plant Biomechanics Group, Freiburg, Germany

*Corresponding author:

hanns-heinz.kassemeyer@wbi.bwl.de (HHK)

Author contributions

KA and HHK concieved the research plans. HHK harvested the fruits. KA performed the chemical analyses. EB, MD and HHK performed the experiments based on cryo-SEM. KA wrote the manuscript with contributions of all authors.

Funding information

This work was partly supported by the European Union program to support cross-border cooperation INTERREG V Upper Rhine 2014-2020 (grant no. 1607) VITIFUTUR - Transnational Platform for Applied Research and Further Training in Viticulture.

Keywords

Berry skin, fruit cuticle, grapevine, epicuticular wax crystals, triterpenoids, very-long-chain aliphatics, *Vitis vinifera*

Abstract

The grapevine berry surface is covered by a cuticle consisting of cutin and various lipophilic wax compounds. The latter build the main barrier for transpirational water loss and protect the fruit against environmental factors e.g. pests, mechanical impacts or radiation. The integrety of the fruit surface is one important key factor for post-harvest quality and storage of fruits. Nonetheless, the developmental pattern of cuticular wax was so far only investigated for a very limited number of fruits. Therefore, we performed comparative investigations on the compositional and morphological nature of epicuticular wax crystals and underlying wax during fruit development in *Vitis vinifera*. The main compound oleanolic acid belongs to the pentacyclic triterpenoids, which occur very early in the development in high amounts inside the cuticle. The amount increases until veraison and decreases further during ripening. In general, very-long chain aliphatic (VLCA) compounds are present in much smaller amounts and alcohols and aldehydes follow the same trend during development. In contrast, the amount of fatty acids constantly increases from fruit set to ripening while wax esters only occur in significant amount at veraison and increase further. Wax crystals at the fruit surface are solely composed of VLCAs and the morphology changes during development according to the compositional changes of the VLCA wax compounds. The remarkable compositional differences between epicuticular wax crystals and the underlying wax are important to understand in terms of studying grape-pest interactions or the influence of environmental factors, since only wax crystals directly face the environment.

Introduction

The outer layer of fleshy fruits, such as berries, fulfils a multiple function as an interface between the fruit containing the seed with the developing embryo and the environment. During the development of the grape berry, the volume increases about 300 times ~~compared to the beginning of berry development~~. In these growth process, the skin of the berries has an important function as a containment of the pericarp since the integrity of the surface must be maintained [1,2]. The berry skin is the outermost, complex structure of the multi-layered exocarp. It consists of a layer of epidermal cells with a thick outer cell wall coated with the cuticle. The matrix of the cuticle is built by a three-dimensional network preferentially composed of polymerized C16 and C18 hydroxy fatty acids [3]. It is impregnated with aliphatic and cyclic wax components. The aliphatic fraction of grape berry wax comprises homologous series of very-long-chain fatty acids and corresponding derivatives such as aldehydes, primary alcohols, alkyl esters and alkanes [4-6]. The cyclic fraction usually contains pentacyclic triterpenoids particularly of the oleanane type [5,7,8]. Some of the wax crystallizes on the outer surface of the cuticle. These epicuticular wax crystals appear as a glaucous covering on the fruit, making the surface extremely water repellent [9,10]. The hydrophobic properties of the wax coating have a crucial function as a preformed barrier to protect the berry from uncontrolled water loss and other environmental factors like pathogens and herbivore attack [11,12]. There is good evidence that the cuticular transpiration barrier is preferentially associated with very-long chain aliphatic wax compounds [3,14], while triterpenoids contribute to the mechanical strength [15], heat stability [16] or biological activity [17] of the cuticle. During berry development, the cuticle has to overcome different challenges depending on the developmental stage. Due to the very rapid volume expansion, it

has to resist strong mechanical stress in order to avoid surface cracking [18]. Therefore, ~~it can be expected that~~ the wax composition is expected to show ~~shows~~ a dynamic developmental pattern. Changes in the chemical-physical properties during berry development can cause stage-specific susceptibility to diseases and pests [19-21). In the same way, cultivar-specific differences in cuticular wax can account ~~be the reason~~ for different susceptibility among grapevine cultivars [22]. Furthermore, fruit wax plays an important role in post-harvest storage processes and fruit quality [11,23] because ~~since~~ the water loss is related to the physico-chemical properties of the cuticular wax [14,24,25]. Here, we performed comparative investigations on the chemical and morphological nature of wax accumulation and the synthesis ~~formation~~ of epicuticular wax crystals during fruit development of two prominent *Vitis vinifera* cultivars ‘Müller Thurgau’ and ‘Blauer Spätburgunder’ also known as ‘Pinot noir’. In a novel approach, we differentiated between the epicuticular wax crystals on the outer surface of the cuticle directly facing the atmosphere and interacting pathogens or insects and the underlying wax ~~which do not come in direct contact with the environment~~. To visualize the highly sensitive micro and nanostructure of the epicuticular wax, we used cryo-scanning electron microscopy. This tool enables to detect finest differences in the structure of the wax crystals which would completely disappear during the fixation process for TEM analysis. A better understanding of the barrier formation during fruit development may help to advance the breeding of more pest resistant *V. vinifera* cultivars, which might also be better adapted to environmental changes.

please reformulate this last sentence because it is quite vague and intangible in a short-term; the scientific outputs of the work are per si very good achievements

Material and Methods

Plant Material

Grape berries of *Vitis vinifera* L. cv. *Müller Thurgau* (MTH) and cv. *Blauer Spätburgunder* (BSB) (also known as *Pinot noir*) were harvested during the growing season 2015 from directly adjacent plots in the experimental vineyards of the Staatliches Weinbauinstitut, Freiburg im Breisgau, Germany. From each plot three plants were randomly selected, from each of which one cluster was taken. ~~According to Lorenz et al. (1994) [26]~~ seven developmental stages from fruit set, were fruits begin to swell (BBCH 71) until berries ripe for harvest (BBCH 89) were investigated (Table 1).

Table 1. Overview of the developmental stages of *Vitis vinifera* cv. Müller Thurgau (MTH) and cv. Blauer Spätburgunder (BSB) berries at the specific sampling dates and the respective fruit diameters; Mean(SD).

					Diameter (mm)	
	Category	Date	BBCH[a]	Description[a]	MTH	BSB
berry formation	1	23.06.2015	71	fruits begin to swell	2.9 (0.3)	3.0 (0.4)
	2	29.06.2015	73	berries groat-sized	5.5 (0.6)	5.4 (0.8)
	3	07.07.2015	75	berries pea sized	9.8 (0.7)	9.8 (1.0)
	4	21.07.2015	77	berries begin to touch	11.9 (0.4)	12.2 (0.4)
veraison	5	04.08.2015	81	beginning of ripening	13.7 (0.4)	13.2 (0.4)
ripening	6	24.08.2015	85	softening of berries	15.7 (0.2)	15.5 (0.3)
	7	21.09.2015	89	berries ripe for harvest	16.5 (0.8)	16.6 (0.4)

[a] Lorenz et al. (1994)

Scanning electron microscopy (Cryo-SEM)

Visualization of the three dimensional surface wax structures was carried out with a scanning-electron microscope (Philips XL30 ESEM) equipped with a cryo preparation unit (Alto 2500, Gatan, UK). For this purpose, three clusters were collected taken early in the morning from the experimental plots ~~of the cultivars to be examined~~. Immediately after sampling, three berries were removed from each grape, from which a slice of the berry skin of approx. separate the units from the number 3mm x 3mm was excised with a scalpel. The slices were mounted with a low temperature glue on a specimen holder by carefully avoiding touching the waxy surface. Cryofixation was performed with nitrogen slush (< -185°C). The frozen samples were sputtered with 20 nm Au in a high vacuum cryo preparation chamber and examined with a SE detector operating with acceleration voltage of 5-10 kV at high vacuum and -150°C. In this way, the specimens were cryo-fixed, sputtered and ready for analysis within 90 min minutes after sampling in the field. A total of nine samples from each variety were analyzed, the surface structure of each specimen was documented from at least three positions at 8.000x magnification.

Wax analysis

Individual berries of the same developmental stage (Table 1) were carefully cut from the bunches by remaining a small piece of the pedicel to prevent leaching from the wound. The berries were washed with deionized water and air-dried. The fruit surface area was determined from the average of the transverse and longitudinal diameter by assuming a spherical shape of the berries. five Fife biological replicates (n = 5) were analyzed with six (category 1) to two (category 7) berries per sample, depending on the developmental stage.

Mechanical removal of epicuticular wax crystals

Small berries up to category 3 were incubated for 5 minutes in a saturated aqueous sodium chloride solution with ultrasonic to mechanically remove the surface wax. Ethanol (10 %) was added to lower the surface tension and ensure a sufficient wetting of the hydrophobic fruit surface. A comparison between treatments with and without ultra-sonic (S1 Fig) showed that epicuticular wax was mechanically grinded off due to the movement of the salt molecules without any chemical extraction, which ensures a separation of the epicuticular wax crystals. After the procedure, berries looked highly polished. As wax crystals became stronger during development, bigger berries from category 4 on were carefully rubbed with tissue until they appeared shiny. The tissue and the aqueous solutions were blended with n-tetracosane as internal standard and extracted three times with chloroform.

Chemical wax extraction

After removal of epicuticular wax crystals, berries were extracted with chloroform for 5 minutes in ultra-sonic. Extracts were washed three times with deionized water to remove hydrophilic organic compounds like sugars. As was described previously, the grape berry wax consist of two fractions, the cyclic triterpenoids and a mixture of long chain aliphatics [27] which were separated with different solvents, to prevent overlying peaks in the gas chromatographic analyzing procedure (S2 Fig). After evaporation to dryness under nitrogen, the residue was dispensed three times in methanol to dissolve the more polar triterpenoid wax fraction. The methanol extracts were filtered and the remaining residue containing the more apolar, aliphatic wax components was dissolved in chloroform. N-tetracosane was added to both fractions as internal standard.

Analytical procedure

All wax samples were evaporated to dryness and hydroxyl-containing compounds were transformed into their corresponding trimethylsilyl derivatives by incubating in bis-*N,O*-(trimethylsilyl)trifluoroacetamide (BSTFA, Macherey-Nagel) in pyridine (Merck) for 30 min at 70° C. The qualitative composition of the wax and the quantity were determined by temperature-controlled capillary gas chromatography and mass spectrometry or flame ionization detection as described earlier [25]. Single compounds were quantified against the internal standard and the amount was normalized to the surface area (µg cm^{-2}). The total amount of wax was calculated by summing up all fractions. Methanol and chloroform fractions were added up and compared to the crystal fraction obtained by mechanical removal.

Results

please change this title according to the legend of the figure

~~Berry development~~

Please remove figure 2 because the growth pattern of berries is well-known - adapt the text of the paragraph

Grape berry development of both cultivars MTH and BSB was almost simultaneous and followed a bi-sigmoidal growth pattern with a rapid increase in size during the first two weeks after berry set (Figs 1and 2) followed by a slightly pronounced lag phase with less growth in July (Figs 1and 2). Upon veraison in the beginning of august, growth resumed (Fig 2), sudden change in color became visible and berries softened (Fig 1 E-G, M-O).

Fig 1. Morphology of grape berries (uppercase) and the respective cryo-scanning electron micrographs (8.000x) of the epicuticular wax crystals (lowercase) of *V. vinifera* cv. Müller Thurgau (left) and cv. Blauer Spätburgunder (right) during development (category 1–7).

~~**Fig 2.** Increase of the berry surface area (cm²) of *V. vinifera* cv. Müller Thurgau (MTH) and cv. Blauer Spätburgunder (BSB) during fruit development (N = 5, Mean ± SD)~~

Morphology of epicuticular wax crystals

In both cultivars, epicuticular wax crystals arised early in the fruit development as a gentle glaucous coating, covering the entire fruit surface (Fig 1 A-D, H-L). When berries please include here the specific phase ~~began to touch each other~~, the wax coat appeared patchier and during maturation, glossy spots became visible (Fig 1 E-G, M-O). The detailed morphological pattern of epicuticular wax crystal formation during fruit development was investigated with Cryo-SEM. At fruit set, were fruits began to swell, cuticular ridges started to spread and the surface in between was covered with a fractured crust according to Barthlott *et al*. (1998)[28]. Underneath the fissures, some primary wax crystals became visible in both cultivars (Fig 1 A, H). One week later (category 2), non-entire parallel stacked platelets protruded perpendicularly from the underlying wax film (Fig 1 B, I). At some sites above ridges, the underlying cuticle or wax film was still visible. During further development, the crystals grew to a dense layer completely covering the cuticle beneath. The basic structure of the wax crystals of mature berries of both varieties studied consists of platelets according to Barthlott et al. (1998) [28] that protrude from the surface at a more or less acute angle. The orientation of the platelets of different heights appears random, resulting in a very irregular pattern. In MTH epicuticular wax appears as membranous platelets with threadlike extensions, which became more distinct during ripening (Figs 3 A and 1 C-G). In BSB the “young” non-entire platelets had rounded margins which became more and more crenated until fruit maturity (Figs 3 B and 1 K-O).

PLEASE CLARIFY THE DIFERENCES BETWEEN THIS FIG AND FIG 1-G AND 1-O, if the result is almost the same, please remove fig 3. or include the results of fig 3 in fig 1 to avoid redundancies

Figure 3 Surface of a berry in full maturity with epicuticular wax crystals recorded with cryo-scanning electron microscope (8.000x). A, In the cultivar Müller-Thurgau epicuticular wax appears as membranous platelets with distinct threadlike extensions. B, Cultivar Blauer Spätburgunder (Pinot noir) shows platelets with rounded margins and on the upper side with crenated edges

remove this figure??

Developmental pattern of total wax

The wax of grape berries of the cultivars MTH and BSB consisted of two fractions (S2 and S3 Figs). The cyclic fraction dominated the entire grape berry wax. Beside the major triterpenoid oleanolic acid also minor amounts of its aldehyde and methyl ester derivatives, erythrodiol, other triterpenoids and several steroids and tocopherols were detected (S2 and S3 Figs). The very-long-chain aliphatics (VLCA) consisted of alcohols (C22-C32), fatty acids (C20-C34), aldehydes (C22-C32) and alkyl esters (C38-C58) predominantly with even chain numbers (S2 and S3 Figs). Alkanes were only found in traces and are therefore not further considered. Beside others, the non-identified fraction included two homologous serious with single diagnostic ions at 280 m/z and 369 m/z and molecular masses in the range of C36-C44 alkyl esters.

The amount and composition of the total cuticular wax was highly dynamic during development. In very young berries (category 1) about 40 µg cm^{-2} triterpenoids were present in the cuticle of both cultivars (Fig 4). The triterpenoid coverage increased significantly to 91.9 ± 3.9 µg cm^{-2} in MTH and 98.0 ± 6.1 µg cm^{-2} in BSB until veraison. From the beginning of august where ripening proceeds (category 5), the triterpenoid coverage per fruit surface area decreased again. At maturation, it reached comparable values as in the beginning of the fruit development (42.3 ± 1.7 µg cm^{-2} in MTH and 51.4 ± 11.7 µg cm^{-2} in BSB, Fig 4), where aliphatics represented

15%

about 15 % of the total wax (S4 Fig). The total amount of aliphatics increased in a similar rate than the triterpenoids, keeping the proportion nearly constant until veraison (S4 Fig). With the beginning of fruit softening (category 5), where triterpenoid coverage decreased, the total aliphatic coverage increased further

5,5% 3.4%

reaching a proportion of 38.4 ± 5.5 % of the total wax in MTH and 35.9 ± 3.4 % in BSB (S4 Fig) at maturity. However, different substance classes of the aliphatics behave different during berry development (Fig 4). In both cultivars the amount of fatty acids, alcohols and aldehydes per surface area increased from the very early fruit development and stagnated or rather decreased again during berry ripening, where a strong increase in alkyl esters was observed (Fig 4). This effect was much more pronounced in MTH where alkyl esters resulted in a final coverage of 16.6 ± 2.7 µg cm^{-2}, which was almost twice as much as in BSB (8.5 ± 0.8 µg cm^{-2}) (Fig. S5). Simultaneously, the quantity of fatty acids and aldehydes at maturity was significantly reduced in MTH compared to BSB (S5 Fig). Detailed information about statistical differences is given in S5 Fig.

Fig 4 Developmental pattern of different substance classes within the total wax (wax crystals plus remaining wax) of *V. vinifera* cv. Müller Thurgau (left) and cv. Blauer

mean

Spätburgunder (right) berries (N = 5, Mean ± SD)

Chemical composition of epicuticular wax crystals

The composition of epicuticular wax crystals and underlying wax was investigated separately by mechanical removal of the outer wax crystals from the surface of grape berries followed by chemical extraction of the remaining wax. The crystals amounted only to a small portion compared to the remaining wax fraction (Figs 5 and 6). They were solely composed of very long chain aliphatics while triterpenoids were almost

completely missing (Fig 5). In very young berries (category 1) only traces of typical wax components were found. From category 2, the load of epicuticular wax crystals increased in both cultivars and was always higher in MTH compared to BSB except for the last stage where it decreased again in MTH (Fig 5). In the beginning of fruit development epicuticular wax crystals were dominated by alcohols (about 76% in MTH, 57% in BSB) and smaller proportions of aldehydes and fatty acids were present. The proportions of alcohols and aldehydes decreased during berry formation and ripening in both cultivars while the proportion of fatty acids increased. At BBCH 75 (category 3) alkyl esters appeared and the proportion increased to about 20% of the total wax crystal composition at maturity in both cultivars (category 7).

Fig 5 Amount of epicuticular wax crystals (µg cm^{-2}) and compositional changes concerning different substance classes during berry development of *V. vinifera* cv. Müller Thurgau (left) and cv. Blauer Spätburgunder (right) (N = 5, Mean ± SD)

Fig 6 Amount (µg cm^{-2}) of remaining wax after mechanical removal of the wax crystals and compositional changes concerning different substance classes during berry development of *V. vinifera* cv. Müller Thurgau (left) and cv. Blauer Spätburgunder (right) (N = 5, Mean ± SD)

~~Remaining wax~~

please improve this title to make more informative

The remaining wax coverage per surface area was much higher than the epicuticular wax crystals. It was dominated by cyclic triterpenoids (Fig 6) with oleanolic acid representing the main portion of about 85 % (MTH1) to 95% (MTH4 and BSB3), depending on the developmental stage. In younger fruits, the portion of aliphatics was about 20%. It increased from veraison (category 5) were alkyl esters appeared in

significant amounts (Fig 6). Upon maturity, alkyl esters comprised 9.8 ± 1.8% of the total intracuticular wax in BSB and 21.9 ± 3.5% in MTH.

Discussion

The overall composition of grape berry cuticular wax is ~~already known since more than~~ has been studied for more than five decades [4,8,27]. However, only one ~~study~~ report compared the chemical plasticity and morphology of the entire cuticular wax (including epi- and intracuticular wax) during grape berry development [29] by using TLC analysis, that allowed only for a crude separation ~~of substance classes~~. Albeit the relative wax composition varies between cultivars, oleanolic acid was reported to be the dominant compound in all investigated grape berry waxes [7,8,30,31]. We also identified oleanolic acid to be the main compound in the total cuticular fruit wax of both investigated *V. vinifera* cultivars, 'Müller Thurgau' and 'Blauer Spätburgunder'. Furthermore, ~~we found~~ remarkable differences were found between the composition of the epicuticular wax bloom and the underlying wax which is partially associated with the cutin matrix. ~~Since~~ Because only the epicuticular wax crystals directly face the environment, it is of high importance to determine the chemical composition separately when dealing e.g. with plant-pest interactions or the impact of radiation. The epicuticular wax crystals, appear early in the fruit development as a glaucous covering on the surface (Fig 1). They totally lack of triterpenoids and are exclusively composed of very-long-chain aliphatic (VLCA) compounds (Fig 5). This pattern was already shown by Possingham et al. (1967) [5] for ripe grape berries of the variety sultana. They found VLCA alcohols to be the main compound beside smaller amounts of acids, esters, aldehydes and hydrocarbons. However, by looking at ripe fruits only a snapshot of the wax composition is mirrored.

please reformulate this sentence; why radiation? what about dehydration, microbiota etc??

please include in the discussion more informative/integrative titles

~~Developmental pattern of cuticular wax~~

Indeed, not only the wax amount but also the composition was shown to be highly dynamic during development. The amount of VLCA fatty acids significantly increases during the fruit development and VLCA alkyl esters occur and increase only later during development (from BBCH 75 on). At any time, triterpenoids are solely located underneath the wax crystals together with additional VLCAs. Khanal et al. (2013) [32] supposed that the stresses and strains that occur during the fruit development as the surface expands are fixed by the current deposition of wax in the cutin, thereby converting reversible elastic into irreversible plastic strain. Additionally, triterpenoids, were shown to be of significant importance for maintaining the mechanical strength of the cuticle [15].

During the later stages, when berry expansion declines, the amount of triterpenoids decrease, as was also previously shown for cherry fruits [33] while the proportion of VLCAs significantly increases (Fig. 4 and S4). From then, substantial amounts of VLCA fatty acids and alkyl esters are deposited in the cuticular wax, leading to significant increase in the proportion of VLCAs up to about 35-40% of the total wax load. VLCA wax compounds rather than triterpenoids are responsible for the permeation barrier in the cuticle [13,14]. During development, triterpenoids are completely shielded against the environment by overlaying VLCAs which also build a dense bloom of wax crystals on the surface (Fig. 1). This became obvious during the experimental procedure. In the beginning, triterpenoids could be extracted by immersing the intact berries in methanol due to their higher polarity compared to VLCAs (personal observation). At developmental stage 3 (BBCH 75) no triterpenoids could be detected any more in methanol extracts of intact fruits since the overlaying VLCAs prevent the methanol from entering the cuticle to extract them. Contrary, in

chloroform extracts, both VLCAs and triterpenoids are found. The chloroform dissolves the VLCAs shielding the outer surface of the cuticle, granting access to the intracuticular triterpenoids. Although, remarkable differences in epi- and intracuticular wax was already shown for leaves [14,34] and fruits [5,35] of several plant species, dissolving fruits or leaves in chloroform is still a very common method to obtain wax

With

from plant surfaces. By using this method, the extracted wax always represents a mixture of epi- and intracuticular wax with variable proportions, depending on the extraction time. This should be considered when the function of wax is related to its

because

chemical composition since the intracuticular wax do not face the environment.

please include in the discussion more informative/integrative titles

Physico-chemical aspects of the wax heterogeneity

this sentence is meaningful

The formation of the layering might be partial self-regulated. The cutin network which is already formed before anthesis [36] might play an important role as stationary phase interacting more or less with the wax molecules, depending on their dimension

proposed (without comma)

or polarity. It was supposed, that the asymmetric cutin matrix has an open branching/network structure with the presence of cavities, which may potentially be occupied by wax molecules [37]. Due to the relatively high polarity of the triterpenoids compared to VLCAs, one would expect that they are captured in the inner layer of the cuticle which is more polar due to the interaction with cell wall polysaccharides. Wide-angle X-ray diffraction and NMR analysis of persimmon fruit cuticles showed that triterpenoids construct a nanocomposite in the cuticular matrix, while VLCAs did not exhibit clear interactions with cutin [15]. They might be transported further via the transpiration stream or simply move by diffusion along a solubility/polarity gradient, finally accumulating as wax film on the surface. Furthermore, the self-assembly process of wax molecules leads to the growth of three-dimensional crystalline

this sentence is meaningful

structures emerging from the underlying wax film [38]. Thereby, the wax chemistry

has a formative influence [39]. In most plant species, the different architectures of the crystals are associated with a single dominating constituent or compound class. While tubules are crystallized from secondary alcohols or ß-diketones, platelets are dominated by primary alcohols, aldehydes or ketones of a certain chain length. In the case of heterogeneous wax mixtures, usually glossy wax films are observed [39,40]. However, in grape berries distinct crystal architecture is observed by SEM despite the fact that we found a heterogeneous mixture of numerous compound classes and chain length. Nevertheless, at fruit set? in the beginning, were alcohols clearly dominate the epicuticular wax (Fig 5) the typical platelets are observed (Fig 1). During berry development, the wax mixture becomes progressively ~~more and~~ more heterogeneous which also affect the crystal structure. With increasing chemical heterogeneity, the initial order yield to an irregular pattern of crenated and fissured wax lobes (Fig 3). In ripe berries, the chemical differences in the epicuticular wax of both cultivars with higher proportions of aldehydes and less alcohols in BSB (Fig 5) is also mirrored by the architecture of the wax crystals. In MTH a separation of the lobes into filamentous structures was strongly pronounced (Fig 3).

wax cristal in fruit defense against pathogen infection??

please make this title more informative

Biological aspects of wax crystal formation

~~The formation of epicuticular wax crystals is of great importance~~. The chemical composition of VLCAs combined with the specific three-dimensional structure resulting from the self-assembly of the wax compounds, leads to a rough super-hydrophobic surface which is very hard-to-wet [41]. Water is repelled very easy and during run off, water droplets will clean the surface from dust and other particles including fungal spores or bacteria [40,42,43], which do not adhere actively to the waxy surface.

Please recenter this sub-chapter specifically on the obtained results; the text should be shortened; as it is, the text is encyclopedic and speculative

infection

It was shown, that the resistance of diverse grape cultivars to the infestation with *Botrytis cinerea* increases with increasing amount of cuticular wax, while the susceptibility of the ripe berries drastically increased at contact sites between berries, where the epicuticular wax was altered [20,21]. We could show that epicuticular wax crystals arise very early in the fruit development shielding the underlying cuticle and intracuticular waxes against the environment within two to three weeks after bloom (BBCH 75). This time window exactly falls together with the susceptibility to the grape powdery mildew *Erysiphe necator* [19] implying that epicuticular wax crystals play an important role in the resistance process. So far it remains unclear, whether the infection success of several fungi is initiated or suppressed by the presence or absence of specific cuticle constituents at different stages of grape berry development. By studying grape-fungus or grape-insect interactions, fundamental differences in the chemical composition of epicuticular wax crystals and the underlying wax have to be considered in the future, since wax crystals completely lacking of triterpenoids are the first contact site between grape berries and pests. ~~Furthermore, a better understanding of physico-chemical aspects in surface associated interactions between plants and their environment may help breeding more resistant plants which are better adapted to environmental challenges.~~

this objective seems intangible, at least in a short term

Acknowledgements

We thank Christine Gernert and Tanja Röder (University of Würzburg) for their technical assistance with GC-MS and GC-FID. We are deeply grateful to Reinhard Jetter (University of Vancouver) for fruitful discussions.

Conflict of interest

All authors declare that there are no conflicts of interest in this work.

References

1. Coombe BG, McCarthy MG. Dynamics of grape berry growth and physiology of ripening. Australian Journal of Grape and Wine Research 2000;6 (2): 131-135.
2. Coombe BG. Research on Development and Ripening of the Grape Berry. American Journal of Enology and Viticulture 1992;43 (1): 101-110.
3. Holloway PJ. The chemical constitution of plant cutins. In The Plant Cuticle, Cutler DF, Alvin, K. L., Price, C. E., Eds. Academic Press: London, 1982, pp 45-85.
4. Radler F, Horn, D. The composition of grape cuticle wax. Australian Journal of Chemistry 1965;18 (7): 1059-1069.
5. Possingham J, Chambers T, Radler F, Grncarevic, M. Cuticular Transpiration and Wax Structure and Composition of Leaves and Fruit of Vitis Vinifera. Australian Journal of Biological Sciences 1967;20 (6): 1149-1154.
6. Shin K, Choi I, Chung K, Lee J, Park H. Morphological development and chemical composition of epicuticular wax crystals in 'Campbell Early' grape. Horticulture, Environment and Biotechnology 2010;51 (4): 253-256.
7. Pensec F, Pączkowski C, Grabarczyk M, Woźniak A, Bénard-Gellon M, Bertsch C, Chong J, Szakiel A. Changes in the Triterpenoid Content of Cuticular Waxes during Fruit Ripening of Eight Grape (*Vitis vinifera*) Cultivars Grown in the Upper Rhine Valley. Journal of Agricultural and Food Chemistry 2014;62 (32): 7998-8007.
8. Radler F. The main Constituents of the Surface Waxes of Varieties and Species of the Genus *Vitis*. American Journal of Enology and Viticulture 1965;16 (3): 159-167.
9. Taylor P. The wetting of leaf surfaces. Current Opinion in Colloid & Interface Science 2011;16 (4): 326-334.
10. Barthlott W, Mail M, Bhushan B, Koch K. Plant Surfaces: Structures and Functions for Biomimetic Innovations. Nano-Micro Letters 2017;9 (2): 23.

11. Lara I, Heredia A, Domínguez E. Shelf Life Potential and the Fruit Cuticle: The Unexpected Player. Frontiers in Plant Science 2019;10 (770).
12. Tang Y, Li Y, Bi Y, Wang Y. Role of Pear Fruit Cuticular Wax and Surface Hydrophobicity in Regulating the Prepenetration Phase of *Alternaria alternata* Infection. Journal of Phytopathology 2017;165 (5): 313-322.
13. Staiger S, Seufert P, Arand K, Burghardt M, Popp C, Riederer M. The permeation barrier of plant cuticles: uptake of active ingredients is limited by very long-chain aliphatic rather than cyclic wax compounds. Pest Management Science 2019;75 (12): 3405-3412.
14. Jetter R, Riederer M. Localization of the Transpiration Barrier in the Epi- and Intracuticular Waxes of Eight Plant Species: Water Transport Resistances Are Associated with Fatty Acyl Rather Than Alicyclic Components. Plant Physiology 2016;170 (2): 921-934.
15. Tsubaki S, Sugimura K, Teramoto Y, Yonemori K, Azuma JI. Cuticular Membrane of Fuyu Persimmon Fruit Is Strengthened by Triterpenoid Nano-Fillers. PLOS ONE 2013;8 (9): e75275.
16. Schuster AC, Burghardt M, Alfarhan A, Bueno A, Hedrich R, Leide J, Thomas J, Riederer M. Effectiveness of cuticular transpiration barriers in a desert plant at controlling water loss at high temperatures. AoB Plants 2016.
17. Szakiel A, Paczkowski C, Huttunen S. Triterpenoid content of berries and leaves of bilberry *Vaccinium myrtillus* from Finland and Poland. J Agric Food Chem 2012;60 (48): 11839-49.
18. Khanal BP, Knoche M. Mechanical properties of cuticles and their primary determinants. Journal of Experimental Botany 2017: 68 (19): 5351-5367.
19. Gee CT, Gadoury DM, Cadle-Davidson L. Ontogenic Resistance to *Uncinula necator* Varies by Genotype and Tissue Type in a Diverse Collection of *Vitis spp*. Plant Disease 2008:92 (7): 1067-1073.
20. Marois JJ, Nelson JK, Morrison JC, Lile LS, Bledsoe AM. The Influence of Berry Contact within Grape Clusters on the Development of *Botrytis cinerea* and Epicuticular Wax. American Journal of Enology and Viticulture 1986;37 (4): 293-296.
21. Percival DC, Sullivan JA, Fisher KH. Effect of cluster exposure, berry contact and cultivar on cuticular membrane formation and occurrence of bunch rot (*Botrytis*

cinerea PERS.: FR.) with 3 *Vitis vinifera* L. cultivars. VITIS - Journal of Grapevine Research 1993;32 (2): 87-97.

22. Gabler FM, Smilanick JL, Mansour, M, Ramming DW, Mackey BE. Correlations of Morphological, Anatomical, and Chemical Features of Grape Berries with Resistance to *Botrytis cinerea*. Phytopathology 2003;93 (10): 1263-1273.
23. Chu W, Gao H, Chen H, Wu W, Fang X. Changes in Cuticular Wax Composition of Two Blueberry Cultivars during Fruit Ripening and Postharvest Cold Storage. Journal of Agricultural and Food Chemistry 2018;66 (11): 2870-2876.
24. Burghardt M, Riederer M. Cuticular Transpiration. In Biology of the plant cuticle, Riederer M, Müller C. Eds. Blackwell Publishing Ltd: 2006, Vol. 23, pp 292-311.
25. Leide J, Hildebrandt U, Reussing K, Riederer M, Vogg G. The developmental pattern of tomato fruit wax accumulation and its impact on cuticular transpiration barrier properties: Effects of a deficiency in a beta-ketoacyl-coenzyme A synthase (LeCER6). Plant Physiology 2007;144 (3): 1667-1679.
26. Lorenz D, Eichhorn K, Bleiholder H, Klose R, Meier U, Weber E. Phänologische Entwicklungsstadien der Weinrebe (*Vitis vinifera* L. ssp. *vinifera*). Codierung und Beschreibung nach der erweiterten BBCH-Skala. Wein-Wissenschaft 1994;49 (2); 66-70.
27. Radler F. The Surface Waxes Of The Sultana Vine (*Vitis vinifera* cv. Thompson Seedless). Australian Journal of Biological Sciences 1965;18: 1045-1065.
28. Barthlott W, Neinhuis C, Cutler D, Ditsch F, Meusel I, Theisen I, Wilhelmi H. Classification and terminology of plant epicuticular waxes. Botanical Journal of the Linnean Society 1998;126 (3); 237-260.
29. Commenil P, Brunet L, Audran JC. The development of the grape berry cuticle in relation to susceptibility to bunch rot disease. Journal of Experimental Botany 1997;48 (8): 1599-1607.
30. Orbán N, Kozák IO, Drávucz M, Kiss A. LC-MS method development to evaluate major triterpenes in skins and cuticular waxes of grape berries. International Journal of Food Science & Technology 2009;44 (4): 869-873.
31. Yamamura H, Naito R. The Surface Wax of Several Grapes in Japan. Journal of the Japanese Society for Horticultural Science 1983;52 (3): 266-272.
32. Khanal BP, Grimm E, Finger S, Blume A, Knoche M. Intracuticular wax fixes and restricts strain in leaf and fruit cuticles. New Phytol 2013;200 (1): 134-43.

33. Peschel S, Franke R, Schreiber L., Knoche M. Composition of the cuticle of developing sweet cherry fruit. Phytochemistry 2007;68 (7): 1017-1025.
34. Buschhaus C, Herz H, Jetter R. Chemical Composition of the Epicuticular and Intracuticular Wax Layers on Adaxial Sides of *Rosa canina* Leaves. Annals of Botany 2007;100 (7): 1557-1564.
35. Vogg G, Fischer S, Leide J, Emmanuel E, Jetter, R, Levy AA, Riederer M. Tomato fruit cuticular waxes and their effects on transpiration barrier properties: functional characterization of a mutant deficient in a very-long-chain fatty acid beta-ketoacyl-CoA synthase. Journal of Experimental Botany 2004:55 (401): 1401-1410.
36. Casado, G, Heredia A. Ultrastructure of the cuticle during growth of the grape berry (*Vitis vinifera*). Physiologia Plantarum 2001;111 (2): 220-224.
37. Fich EA, Segerson NA, Rose J K C. The Plant Polyester Cutin: Biosynthesis, Structure, and Biological Roles. Annual Review of Plant Biology 2016, 67 (1): 207-233.
38. Koch K, Neinhuis, C, Ensikat HJ. Barthlott W. Self assembly of epicuticular waxes on living plant surfaces imaged by atomic force microscopy (AFM). Journal of Experimental Botany 2004;55 (397): 711-718.
39. Koch K, Ensikat HJ. The hydrophobic coatings of plant surfaces: Epicuticular wax crystals and their morphologies, crystallinity and molecular self-assembly. Micron 2008;39 (7): 759-772.
40. Bargel H, Koch K, Cerman Z. Neinhuis C. Evans Review No. 3: Structure–function relationships of the plant cuticle and cuticular waxes — a smart material? Functional Plant Biology 2006;33 (10): 893-910.
41. Barthlott W, Mail M, Neinhuis C. Superhydrophobic hierarchically structured surfaces in biology: evolution, structural principles and biomimetic applications. Philosophical Transactions of the Royal Society of London A: Mathematical, Physical and Engineering Sciences 2016;374 (2073).
42. Neinhuis C, Barthlott W. Characterization and Distribution of Water-repellent, Self-cleaning Plant Surfaces. Annals of Botany 1997:79 (6): 667-677.
43. Koch K, Barthlott W. Superhydrophobic and superhydrophilic plant surfaces: an inspiration for biomimetic materials. Philos Trans A Math Phys Eng Sci 2009;367: 1487-1509.

Supporting Information

S1 Fig. Comparison of chromatographs (GC-FID) for different extraction methods of epicuticular waxes of *Vitis vinifera* cv. Müller Thurgau berries (category 3). 1: deionized water with 5 min ultra-sonic, 2: 10% ethanol in saturated aqueous sodium chloride solution without ultra-sonic, 3: 10% ethanol in saturated aqueous sodium chloride solution with 5 min ultra-sonic. IS: internal standard n-tetracosane (3 µg).

S2. Fig Comparison of chromatographs (GC-FID) for different extraction methods of epicuticular wax crystals (mechanical removal) and the underlying wax (MeOH and $CHCl_3$ extraction) of *Vitis vinifera* cv. Müller Thurgau berries (category 3). IS: internal standard n-tetracosane added in different amounts (mechanical removal: 5 µg, MeOH extraction: 25 µg, $CHCl_3$ extraction: 10 µg).

S3 Fig. Detailed composition of the total wax (epi- plus intracuticular) of three representative developmental stages (1, 4, 7) of *Vitis vinifera* cv. Müller Thurgau berries. The numbers on the x-Axis refer to the carbon chain length of the aliphatic compounds. (N=5, Mean + SD)

S4 Fig. Detailed composition of the total wax (epi- plus intracuticular) of three representative developmental stages (1, 4, 7) of *Vitis vinifera* cv. Blauer Spätburgunder berries. The numbers on the x-Axis refer to the carbon chain length of the aliphatic compounds. (N=5, Mean + SD)

S5 Fig. Proportional change of cyclic and aliphatic fractions in the total cuticular wax of *Vitis vinifera* cv. Müller Thurgau (MTH) and cv. Blauer Spätburgunder (BSB) berries during fruit development. (N=5, Mean ± SD)

S6 Fig. Developmental changes in the coverage (µg cm^{-2}) of individual substance classes within the total wax of *Vitis vinifera* cv. Müller Thurgau (MTH) and cv. Blauer Spätburgunder (BSB) berries. Two-way analysis of variance was performed within

substance classes and all pairwise multiple comparison procedure (Holm-Sidak method) was used to detect significant differences (indicated by different letters) within cultivars during development (MTH: uppercase, BSB: lowercase) and between both cultivars at a given developmental stage (*: $p < 0.05$, **: $p < 0.001$, DF = 66, Mean + SD).

Figure Legends

Figure 1 Morphology of grape berries (uppercase) and the respective epicuticular wax crystals (lowercase) of *V. vinifera* cv. Müller Thurgau (left) and cv. Blauer Spätburgunder (right) during development (category 1–7). Cryo-scanning electron micrographs of the epicuticular wax structures

Figure 2 Increase of the berry surface area (cm^2) of *V. vinifera* cv. Müller Thurgau (MTH) and cv. Blauer Spätburgunder (BSB) during fruit development (N = 5, Mean ± SD)

Figure 3 Surface of a berry in full maturity with epicuticular wax crystals recorded with cryo-scanning electron microscope. A, In the cultivar Müller-Thurgau epicuticular wax appears as membranous platelets with distinct threadlike extensions. B, Cultivar Blauer Spätburgunder (Pinot noir) shows platelets with rounded margins and on the upper side with crenated edges

Figure 4 Developmental pattern of different substance classes within the total wax (wax crystals plus remaining wax) of *V. vinifera* cv. Müller Thurgau (left) and cv. Blauer Spätburgunder (right) berries (N = 5, Mean ± SD)

Figure 5 Amount of epicuticular wax crystals (µg cm^{-2}) and compositional changes concerning different substance classes during berry development of *V. vinifera* cv. Müller Thurgau (left) and cv. Blauer Spätburgunder (right) (N = 5, Mean ± SD)

Figure 6 Amount (µg cm^{-2}) of remaining wax after mechanical removal of the wax crystals and compositional changes concerning different substance classes during berry development of *V. vinifera* cv. Müller Thurgau (left) and cv. Blauer Spätburgunder (right) (N = 5, Mean ± SD)

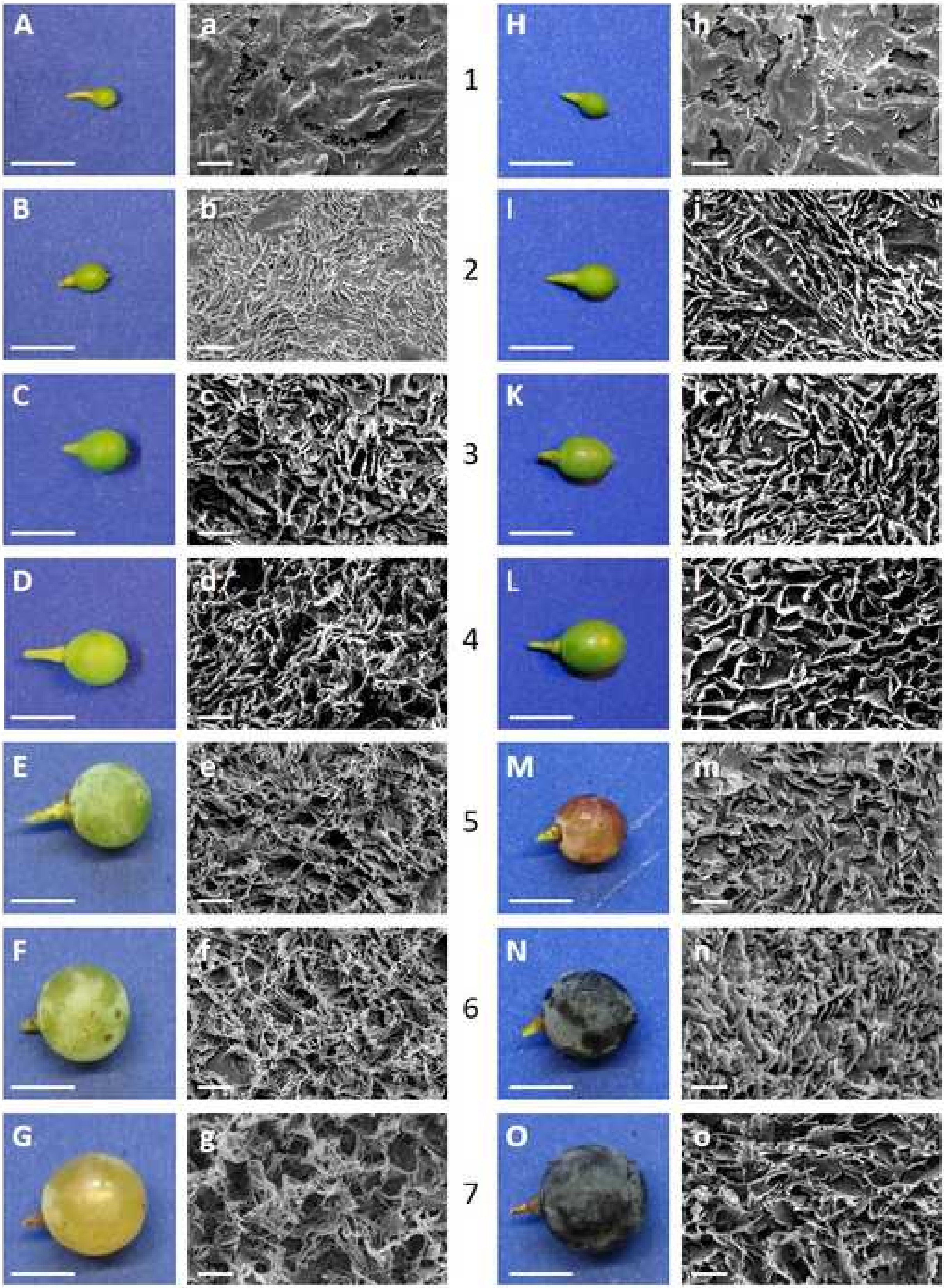
A
a
1
H
h
B
b
2
I
i
C
c
3
K
k
D
d
4
L
l
E
e
5
M
m
F
f
6
N
n
G
g
7
O
o

Click here to access/download;Figure;Fig2.tif

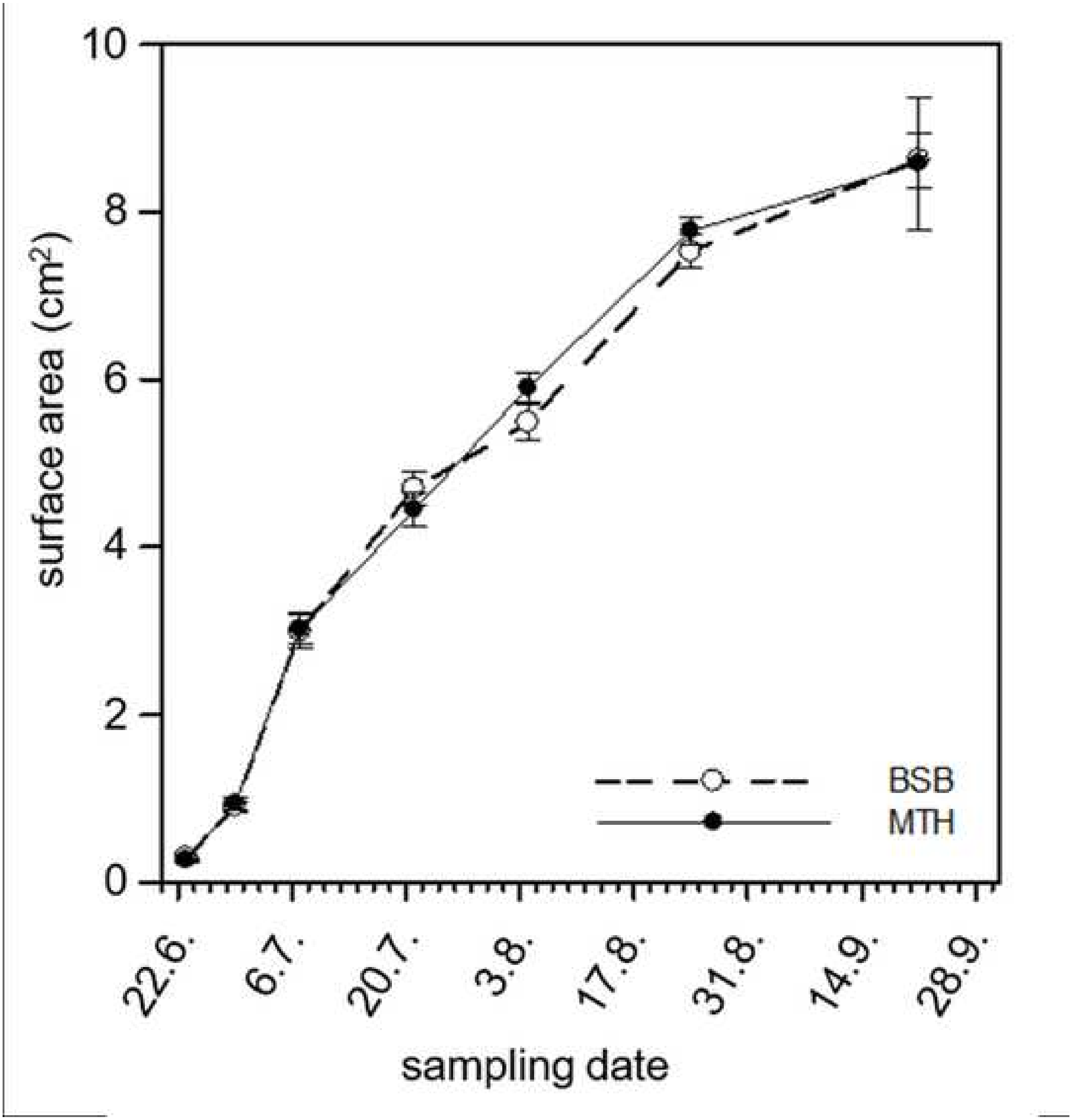

Click here to access/download;Figure;Fig3.tif

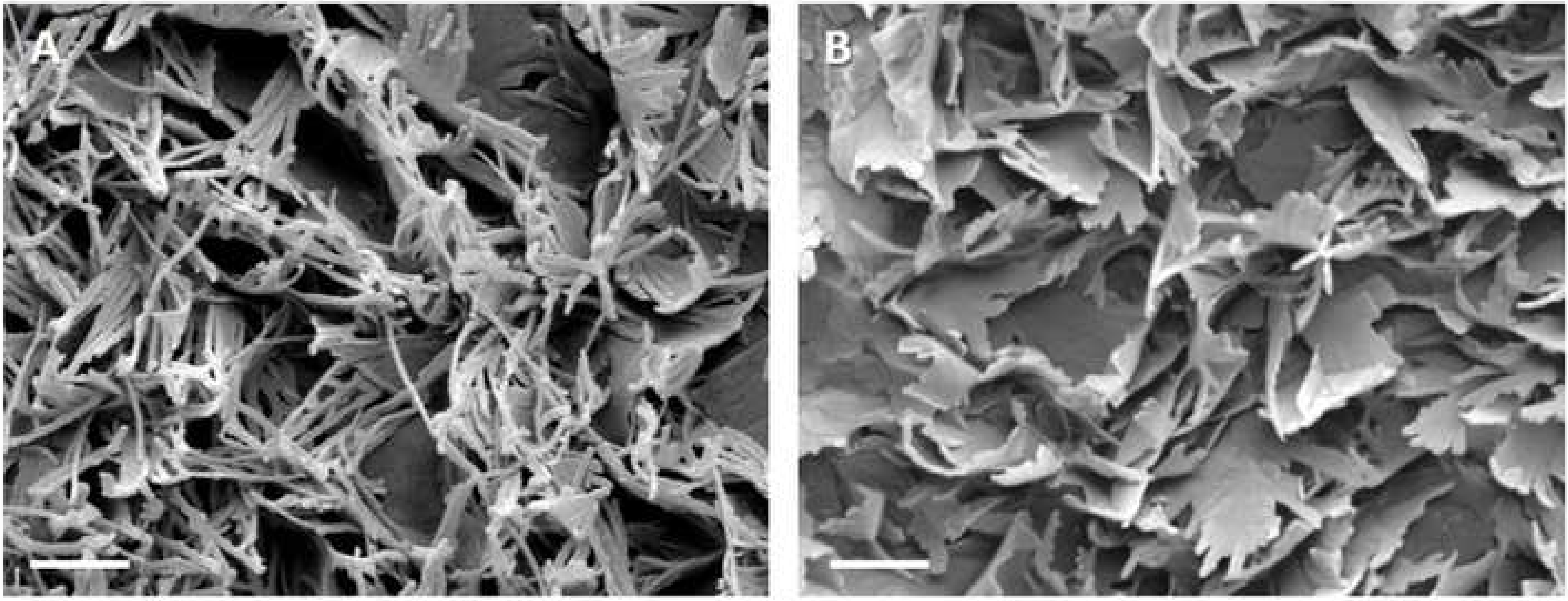

Click here to access/download;Figure;Fig4.tif

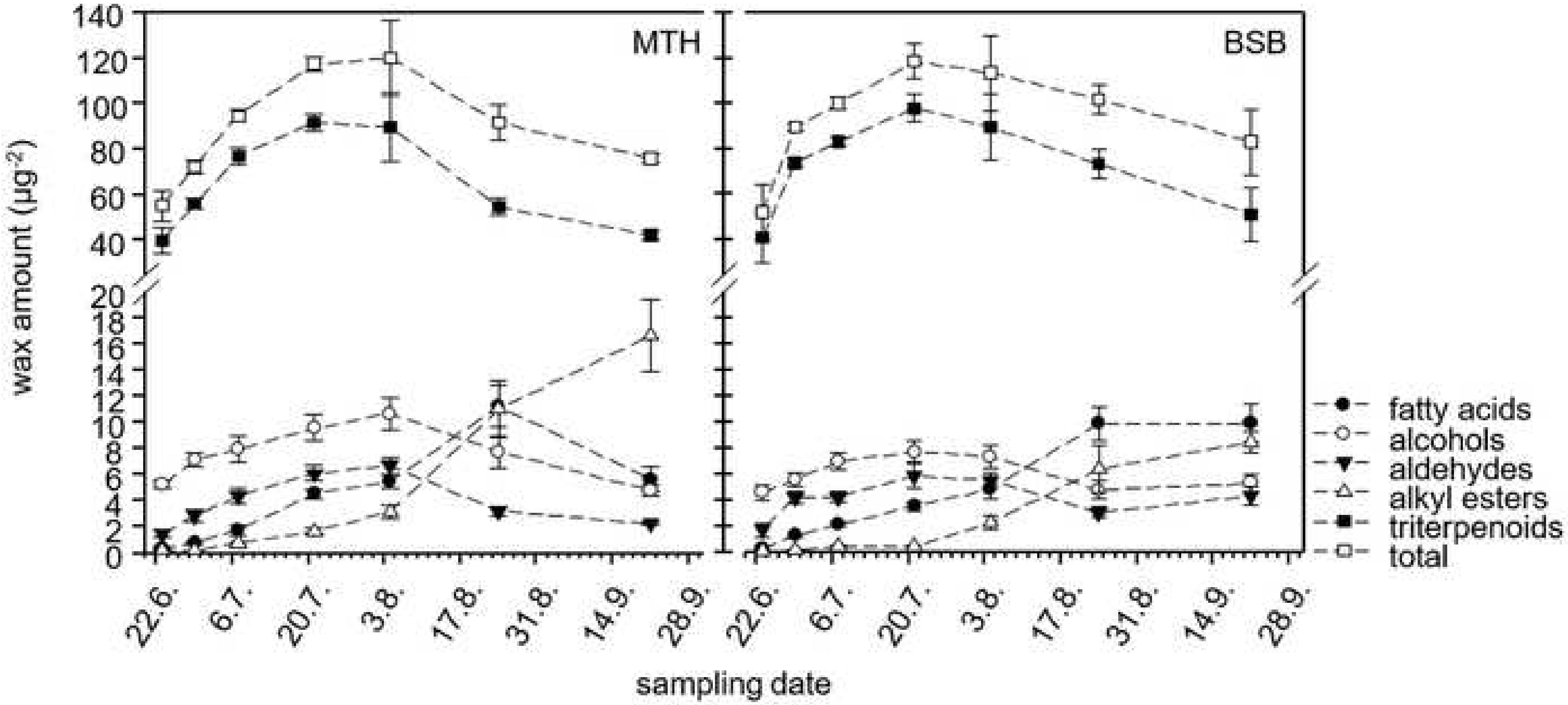

Click here to access/download;Figure;Fig5.tif

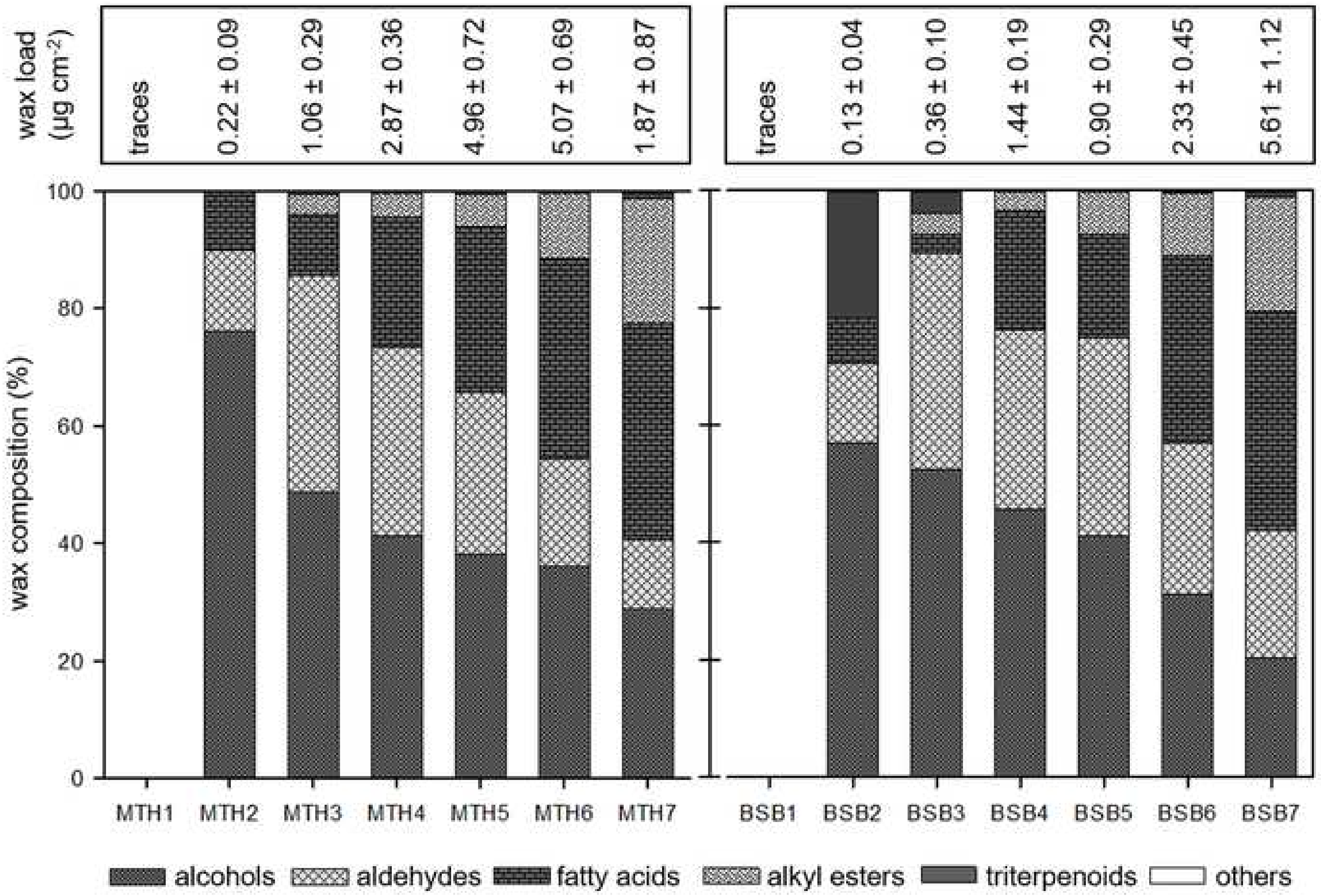

Click here to access/download;Figure;Fig6.tif

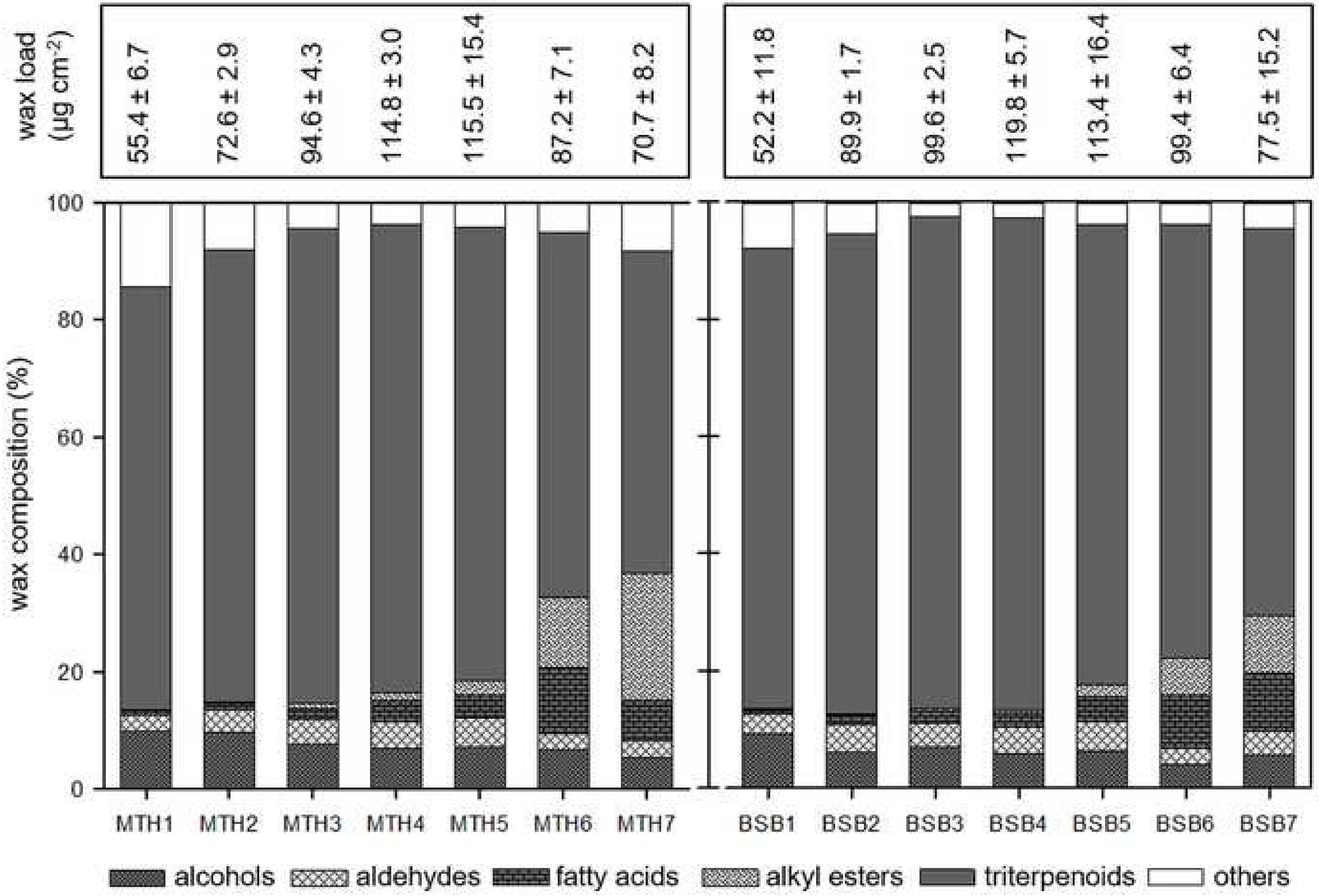

Click here to access/download
Supporting Information
Figure S1.tif

Click here to access/download
Supporting Information
Figure S2.tif

Click here to access/download
Supporting Information
Figure S3.tif

Click here to access/download
Supporting Information
Figure S4.tif

Click here to access/download
Supporting Information
Supplement Fig. S5.tif

Click here to access/download
Supporting Information
Supplement Fig. S6.tif
