# Supplementary material for: Developmental pattern of grapevine (Vitis vinifera L.) berry cuticular wax: Differentiation between epicuticular crystals and underlying wax
Source: PLoS One. 2021 Feb 19;16(2):e0246693. doi: 10.1371/journal.pone.0246693 (PMC7894928; doi:10.1371/journal.pone.0246693)
Supplement: Attachment — Submitted filename: Response to reviewer.docx [file pone.0246693.s008.docx]

Prof. Dr. Hanns-Heinz Kassemeyer

State Institute for Viticulture Freiburg Merzhauser Str. 119

79100 Freiburg Germany

hanns-heinz.kassemeyer@wbi.bwl.de

Wednesday, 13 January 2021

Dear reviewers,

First of all, thank you for reviewing our manuscript "Developmental pattern of grapevine (Vitis vinifera L.) berry cuticular wax: differentiation between epicuticular crystals and underlying wax" and the very helpful suggestions and corrections for improvement. We have taken into account most of the changes and amendments you suggested. Please find below comments on these revised positions.

**Reviewer#1**

*Line 71 “pentacyclic triterpenoids particularly of the oleanane type”; please add also ursane (i.e., particularly of the oleanane and ursane type). Some berries contain ursolic acid as the main constituent in cuticular waxes (e,g., lingonberry, highbush blueberry etc.), besides, many fruits contain the mixture of acids of both types.*

p4 line 71-73

I agree that ursane type needs to be added as it is also an important component of cuticular waxes in berries. I have added ursane-type so that now reads as you suggested: “…particularly of the oleanane and ursane type..”.

*Line 157. “After removal of epicuticular wax crystals, berries were extracted with chloroform for 5 minutes in ultra-sonic. Extracts were washed three times with deionized water to remove hydrophilic organic compounds like sugars”.
The time of extraction is relatively long, this method may cause some contamination with constituents present in deeper layer (I don’t mention sugars but triterpenoids that can be present in other tissues.) Have the Authors tried to validate this method in the context of the depth of chloroform penetration? I am not critizing this method as not suitable, I am just wondering what was the reason of choice of this time of extraction.*

p 8 line 162

Deviating from the standard method with 30 sec extraction time, we used the optimal extraction time of 5 min for berries according to our experience. In this way, we reduced large variations in the wax content of the extracts. We have been using this extraction time, which deviates from the standard method, in our laboratory for quite some time and have successfully validated it in many experiments.

**Reviewer#2**

*Material & Methods. I would like to invite authors to mention in the material and method section, the position of selected clusters within the canopy as well as the position within the cluster of selected berries, since cuticle is highly dependent on cluster microclimate (e.g. temperature, humidity and radiation).*

I fully agree that the microclimate in the canopy and inside the cluster can lead to deviations in the wax composition. Therefore, we have taken care of this during sampling. and in the descriptions of the sampling pointed out: “For this purpose, three intact clusters inserted between the second and third nodes of the shoot were collected early in the morning from the experimental plots. Immediately after sampling, three berries of the same size were removed from the inside of each cluster….”

*p 5, line 100-103 Please reformulate this last sentences (Introduction) because it is quite vague and intangible in a short-term; the scientific outputs of the work are per se very good achievements.*

We reformulated the last sentence of the introduction to clearly articulate the outcome of the work and replaced it by: „The scientific results of this work represent significant achievements for targeted breeding of grapevine cultivars with resistance to pests and pathogens and higher berry resilience to environmental changes”.

*Results Berry development. Please change this title according to the legend of the figure*

The first two paragraphs have been combined and we improved the heading of the paragraph to make it more informative.

*Results Berry development. Please remove figure 2 because the growth pattern of berries is well-known.*

We removed Fig 2 showing the berry growth curve.

*Results Berry development.* *Please clarify the differences between Tis Fig (Fig 3) and Fig1 1-G and 1-O, if the result is almost the same, please remove fig 3. or include the results of fig 3 in fig 1 to avoid redundancies.*

We kept this Fig (now Fig 2), because it shows the structure of the wax crystals of the two cultivars in detail. In the discussion (p 18, line 396), we explain the different structure of the wax crystals of the two varieties, which can be seen very clearly in the two images.

*Results Remaining wax 13. Please remove this title to make more informative*

We changed the titles of all paragraphs of the results to make them more informative.

*Discussion p 15-18 Please include in the discussion more informative/integrative titles.*

As with the results, we improved the headings of the paragraphs in the discussion to make them more infomative.

*Discussion p 14 line 303-306. Please reformulate this sentence (“Since only the epicuticular wax crystals directly face the environment, it is of high importance to determine the chemical composition separately when dealing e.g. with plant-pest interactions or the impact of radiation.”); why radiation? What about dehydration, microbiota etc??*

We reformulated these sentences now reading: “The epiculticular wax on the surface represent the interface between the berry and the environment. Therefore, the chemical and structural properties of this boundary layer are highly important for coping with abiotic and biotic external influences such as UV-radiation, dehydration, pests and pathogens.”

*Discussion p 19 Biological aspects of wax crystal formation. Please recenter this sub-chapter specifically on the obtained results; the text should be shortened; as it is, the text is encyclopedic and speculative.*

We shortened the last paragraph of the discussion and matched it to the statement in the last sentence of the introduction.

*I also invite authors to check errors in spelling along the text due to tape mistakes, like in line 140, ‘five’ instead of ‘fife’.*

We have corrected all the spelling errors in the text that reviewer 2 has kindly noted

Please note that as a result of the revision, the line numbers of the originally submitted manuscript have changed.

Hopefully, with these corrections and amendments, we have now improved our manuscript sufficiently for publication. Again, thank you for your efforts.

With my best regards

Hanns-Heinz Kassemeyer
