# Supplementary material for: Developmental pattern of grapevine (Vitis vinifera L.) berry cuticular wax: Differentiation between epicuticular crystals and underlying wax
Source: PLoS One. 2021 Feb 19;16(2):e0246693. doi: 10.1371/journal.pone.0246693 (PMC7894928; doi:10.1371/journal.pone.0246693)
Supplement: Peer review history

# Peer review: Decision Letter 0

**DOI:** 10.1371/journal.pone.0246693.r001

**Authors:** Hernâni Gerós

**Transfer Alert**

This paper was transferred from another journal. As a result, its full editorial history (including decision letters, peer reviews and author responses) may not be present.

1 Dec 2020

PONE-D-20-32426

Developmental pattern of grapevine (Vitis vinifera L.) berry cuticular wax: differentiation between epicuticular crystals and underlying wax

PLOS ONE

Dear Dr. Kassemeyer,

Thank you for submitting your manuscript to PLOS ONE. After careful consideration, we feel that it has merit but does not fully meet PLOS ONE’s publication criteria as it currently stands. Therefore, we invite you to submit a revised version of the manuscript that addresses the minor points raised during the review process. Please see below the comments of both reviewers that were very positive and constructive.

I also agree that your manuscript is scientifically very relevant. Please, to prepare the final version take into consideration the suggestions and recommendations I included in the attached pdf.

Please submit your revised manuscript by Jan 15 2021 11:59PM. If you will need more time than this to complete your revisions, please reply to this message or contact the journal office at plosone@plos.org. When you're ready to submit your revision, log on to https://www.editorialmanager.com/pone/ and select the 'Submissions Needing Revision' folder to locate your manuscript file.

Please include the following items when submitting your revised manuscript:

- A rebuttal letter that responds to each point raised by the academic editor and reviewer(s). You should upload this letter as a separate file labeled 'Response to Reviewers'.
- A marked-up copy of your manuscript that highlights changes made to the original version. You should upload this as a separate file labeled 'Revised Manuscript with Track Changes'.
- An unmarked version of your revised paper without tracked changes. You should upload this as a separate file labeled 'Manuscript'.

If you would like to make changes to your financial disclosure, please include your updated statement in your cover letter. Guidelines for resubmitting your figure files are available below the reviewer comments at the end of this letter.

If applicable, we recommend that you deposit your laboratory protocols in protocols.io to enhance the reproducibility of your results. Protocols.io assigns your protocol its own identifier (DOI) so that it can be cited independently in the future. For instructions see: http://journals.plos.org/plosone/s/submission-guidelines#loc-laboratory-protocols

We look forward to receiving your revised manuscript.

Kind regards,

Hernâni Gerós, PhD

Academic Editor

PLOS ONE

Journal Requirements:

When submitting your revision, we need you to address these additional requirements.

1. Please ensure that your manuscript meets PLOS ONE's style requirements, including those for file naming. The PLOS ONE style templates can be found at

https://journals.plos.org/plosone/s/file?id=wjVg/PLOSOne_formatting_sample_main_body.pdf and

https://journals.plos.org/plosone/s/file?id=ba62/PLOSOne_formatting_sample_title_authors_affiliations.pdf

2. Thank you for stating in your Funding Statement:

'This work was partly supported by the European Union program to support cross-border cooperation INTERREG V Upper Rhine 2014-2020 (grant no. 1607) VITIFUTUR - Transnational Platform for Applied Research and Further Training in Viticulture.'

a. Please provide an amended statement that declares *all* the funding or sources of support (whether external or internal to your organization) received during this study, as detailed online in our guide for authors at http://journals.plos.org/plosone/s/submit-now

Please also include the statement “There was no additional external funding received for this study.” in your updated Funding Statement.

b. Please include your amended Funding Statement within your cover letter. We will change the online submission form on your behalf.

[Note: HTML markup is below. Please do not edit.]

Reviewers' comments:

Reviewer's Responses to Questions

**Comments to the Author**

1. Is the manuscript technically sound, and do the data support the conclusions?

The manuscript must describe a technically sound piece of scientific research with data that supports the conclusions. Experiments must have been conducted rigorously, with appropriate controls, replication, and sample sizes. The conclusions must be drawn appropriately based on the data presented. 

Reviewer #1: Yes

Reviewer #2: Yes

**********

2. Has the statistical analysis been performed appropriately and rigorously? 

Reviewer #1: Yes

Reviewer #2: Yes

**********

3. Have the authors made all data underlying the findings in their manuscript fully available?

The PLOS Data policy requires authors to make all data underlying the findings described in their manuscript fully available without restriction, with rare exception (please refer to the Data Availability Statement in the manuscript PDF file). The data should be provided as part of the manuscript or its supporting information, or deposited to a public repository. For example, in addition to summary statistics, the data points behind means, medians and variance measures should be available. If there are restrictions on publicly sharing data—e.g. participant privacy or use of data from a third party—those must be specified.

Reviewer #1: Yes

Reviewer #2: Yes

**********

4. Is the manuscript presented in an intelligible fashion and written in standard English?

PLOS ONE does not copyedit accepted manuscripts, so the language in submitted articles must be clear, correct, and unambiguous. Any typographical or grammatical errors should be corrected at revision, so please note any specific errors here.

Reviewer #1: Yes

Reviewer #2: Yes

**********

5. Review Comments to the Author

Please use the space provided to explain your answers to the questions above. You may also include additional comments for the author, including concerns about dual publication, research ethics, or publication ethics. (Please upload your review as an attachment if it exceeds 20,000 characters)

Reviewer #1: The development of plant cuticle involving the morphological and chemical changes occurring on the fruit surface during fruit growth and maturation is a very important phenomenon, crucial for features of the fruit quality. The study presented in the manuscript concerns the processes which take place in cuticular waxes of grape berry (the fruit of Vinis vinifera). In many countries, grape is economically one of the most important crops plants, so this study is particularly interesting in the context of grape resistance to pathogens as well as various environmental stresses. The results illustrating the compositional differences between epicuticular wax crystals and the underlying wax are new, original and they supplement our knowledge of plant cuticle and the role of cuticular waxes.

The manuscript is well written and scientifically sound. The experiments were carefully planned and properly performed. The methods are modern and suitable for the aim of the study. I consider this manuscript as very valuable and worth publishing.

Some small comments:

Line 71 “pentacyclic triterpenoids particularly of the oleanane type”; please add also ursane (i.e., particularly of the oleanane and ursane type). Some berries contain ursolic acid as the main constituent in cuticular waxes (e,g., lingonberry, highbush blueberry etc.), besides, many fruits contain the mixture of acids of both types.

Line 157. “After removal of epicuticular wax crystals, berries were extracted with chloroform for 5 minutes in ultra-sonic. Extracts were washed three times with deionized water to remove hydrophilic organic compounds like sugars”.

The time of extraction is relatively long, this method may cause some contamination with constituents present in deeper layer (I don’t mention sugars but triterpenoids that can be present in other tissues.) Have the Authors tried to validate this method in the context of the depth of chloroform penetration? I am not critizing this method as not suitable, I am just wondering what was the reason of choice of this time of extraction.

Reviewer #2: The study presented interesting results related to the formation and composition of the different layers of cuticle in a highly important cultivated fruit crop, winegrapes. Particularly this investigation shows for the first time in my knowledge, metabolic differences between epiculticular crystals and underlying waxes in berries, which is of great importance at the moment to undertake investigations related to biotic and abiotic cues on fruit surface protection.

The manuscript is well organized and well written with consistent results and adequate discussion. However, I would like to invite authors to mention in the material and method section, the position of selected clusters within the canopy as well as the position within the cluster of selected berries, since cuticle is highly dependent on cluster microclimate (e.g. temperature, humidity and radiation).

I also invite authors to check errors in spelling along the text due to tape mistakes, like in line 140, ‘five’ instead of ‘fife’.

**********

6. PLOS authors have the option to publish the peer review history of their article (what does this mean?). If published, this will include your full peer review and any attached files.

If you choose “no”, your identity will remain anonymous but your review may still be made public.

**Do you want your identity to be public for this peer review?** For information about this choice, including consent withdrawal, please see our Privacy Policy.

Reviewer #1: No

Reviewer #2: **Yes: **Olfa Zarrouk

[NOTE: If reviewer comments were submitted as an attachment file, they will be attached to this email and accessible via the submission site. Please log into your account, locate the manuscript record, and check for the action link "View Attachments". If this link does not appear, there are no attachment files.]

While revising your submission, please upload your figure files to the Preflight Analysis and Conversion Engine (PACE) digital diagnostic tool, https://pacev2.apexcovantage.com/. PACE helps ensure that figures meet PLOS requirements. To use PACE, you must first register as a user. Registration is free. Then, login and navigate to the UPLOAD tab, where you will find detailed instructions on how to use the tool. If you encounter any issues or have any questions when using PACE, please email PLOS at figures@plos.org. Please note that Supporting Information files do not need this step.

Attachment Submitted filename: PONE-D-20-32426.HG.pdf Click here for additional data file.

# Author comment: Author response to Decision Letter 0

**DOI:** 10.1371/journal.pone.0246693.r002

13 Jan 2021

Dear reviewers,

First of all, thank you for reviewing our manuscript "Developmental pattern of grapevine (Vitis vinifera L.) berry cuticular wax: differentiation between epicuticular crystals and underlying wax" and the very helpful suggestions and corrections for improvement. We have taken into account most of the changes and amendments you suggested. Please find below comments on these revised positions.

Reviewer#1

Line 71 “pentacyclic triterpenoids particularly of the oleanane type”; please add also ursane (i.e., particularly of the oleanane and ursane type). Some berries contain ursolic acid as the main constituent in cuticular waxes (e,g., lingonberry, highbush blueberry etc.), besides, many fruits contain the mixture of acids of both types.

p4 line 71-73

I agree that ursane type needs to be added as it is also an important component of cuticular waxes in berries. I have added ursane-type so that now reads as you suggested: “…particularly of the oleanane and ursane type..”.

Line 157. “After removal of epicuticular wax crystals, berries were extracted with chloroform for 5 minutes in ultra-sonic. Extracts were washed three times with deionized water to remove hydrophilic organic compounds like sugars”.

The time of extraction is relatively long, this method may cause some contamination with constituents present in deeper layer (I don’t mention sugars but triterpenoids that can be present in other tissues.) Have the Authors tried to validate this method in the context of the depth of chloroform penetration? I am not critizing this method as not suitable, I am just wondering what was the reason of choice of this time of extraction.

p 8 line 162

Deviating from the standard method with 30 sec extraction time, we used the optimal extraction time of 5 min for berries according to our experience. In this way, we reduced large variations in the wax content of the extracts. We have been using this extraction time, which deviates from the standard method, in our laboratory for quite some time and have successfully validated it in many experiments.

 

Reviewer#2

Material & Methods. I would like to invite authors to mention in the material and method section, the position of selected clusters within the canopy as well as the position within the cluster of selected berries, since cuticle is highly dependent on cluster microclimate (e.g. temperature, humidity and radiation).

I fully agree that the microclimate in the canopy and inside the cluster can lead to deviations in the wax composition. Therefore, we have taken care of this during sampling. and in the descriptions of the sampling pointed out: “For this purpose, three intact clusters inserted between the second and third nodes of the shoot were collected early in the morning from the experimental plots. Immediately after sampling, three berries of the same size were removed from the inside of each cluster….”

p 5, line 100-103 Please reformulate this last sentences (Introduction) because it is quite vague and intangible in a short-term; the scientific outputs of the work are per se very good achievements.

We reformulated the last sentence of the introduction to clearly articulate the outcome of the work and replaced it by: „The scientific results of this work represent significant achievements for targeted breeding of grapevine cultivars with resistance to pests and pathogens and higher berry resilience to environmental changes”.

Results Berry development. Please change this title according to the legend of the figure

The first two paragraphs have been combined and we improved the heading of the paragraph to make it more informative.

Results Berry development. Please remove figure 2 because the growth pattern of berries is well-known.

We removed Fig 2 showing the berry growth curve.

Results Berry development. Please clarify the differences between Tis Fig (Fig 3) and Fig1 1-G and 1-O, if the result is almost the same, please remove fig 3. or include the results of fig 3 in fig 1 to avoid redundancies.

We kept this Fig (now Fig 2), because it shows the structure of the wax crystals of the two cultivars in detail. In the discussion (p 18, line 396), we explain the different structure of the wax crystals of the two varieties, which can be seen very clearly in the two images.

Results Remaining wax 13. Please remove this title to make more informative

We changed the titles of all paragraphs of the results to make them more informative.

 

Discussion p 15-18 Please include in the discussion more informative/integrative titles.

As with the results, we improved the headings of the paragraphs in the discussion to make them more infomative.

Discussion p 14 line 303-306. Please reformulate this sentence (“Since only the epicuticular wax crystals directly face the environment, it is of high importance to determine the chemical composition separately when dealing e.g. with plant-pest interactions or the impact of radiation.”); why radiation? What about dehydration, microbiota etc??

We reformulated these sentences now reading: “The epiculticular wax on the surface represent the interface between the berry and the environment. Therefore, the chemical and structural properties of this boundary layer are highly important for coping with abiotic and biotic external influences such as UV-radiation, dehydration, pests and pathogens.” 

Discussion p 19 Biological aspects of wax crystal formation. Please recenter this sub-chapter specifically on the obtained results; the text should be shortened; as it is, the text is encyclopedic and speculative.

We shortened the last paragraph of the discussion and matched it to the statement in the last sentence of the introduction.

I also invite authors to check errors in spelling along the text due to tape mistakes, like in line 140, ‘five’ instead of ‘fife’.

We have corrected all the spelling errors in the text that reviewer 2 has kindly noted

Please note that as a result of the revision, the line numbers of the originally submitted manuscript have changed.

Hopefully, with these corrections and amendments, we have now improved our manuscript sufficiently for publication. Again, thank you for your efforts.

With my best regards

Hanns-Heinz Kassemeyer

Attachment Submitted filename: Response to reviewer.docx Click here for additional data file.

# Editor report: Decision Letter 1

**DOI:** 10.1371/journal.pone.0246693.r003

**Authors:** Hernâni Gerós

25 Jan 2021

Developmental pattern of grapevine (Vitis vinifera L.) berry cuticular wax: differentiation between epicuticular crystals and underlying wax

PONE-D-20-32426R1

Dear Dr. Kassemeyer,

We’re pleased to inform you that your manuscript has been judged scientifically suitable for publication and will be formally accepted for publication once it meets all outstanding technical requirements.

Within one week, you’ll receive an e-mail detailing the required amendments. When these have been addressed, you’ll receive a formal acceptance letter and your manuscript will be scheduled for publication.

An invoice for payment will follow shortly after the formal acceptance. To ensure an efficient process, please log into Editorial Manager at http://www.editorialmanager.com/pone/, click the 'Update My Information' link at the top of the page, and double check that your user information is up-to-date. If you have any billing related questions, please contact our Author Billing department directly at authorbilling@plos.org.

If your institution or institutions have a press office, please notify them about your upcoming paper to help maximize its impact. If they’ll be preparing press materials, please inform our press team as soon as possible -- no later than 48 hours after receiving the formal acceptance. Your manuscript will remain under strict press embargo until 2 pm Eastern Time on the date of publication. For more information, please contact onepress@plos.org.

Kind regards,

Hernâni Gerós, PhD

Academic Editor

PLOS ONE

Additional Editor Comments (optional):

Reviewers' comments:

# Editor report: Acceptance letter

**DOI:** 10.1371/journal.pone.0246693.r004

**Authors:** Hernâni Gerós

2 Feb 2021

PONE-D-20-32426R1 

Developmental pattern of grapevine (*Vitis vinifera* L.) berry cuticular wax: differentiation between epicuticular crystals and underlying wax 

Dear Dr. Kassemeyer:

I'm pleased to inform you that your manuscript has been deemed suitable for publication in PLOS ONE. Congratulations! Your manuscript is now with our production department. 

If your institution or institutions have a press office, please let them know about your upcoming paper now to help maximize its impact. If they'll be preparing press materials, please inform our press team within the next 48 hours. Your manuscript will remain under strict press embargo until 2 pm Eastern Time on the date of publication. For more information please contact onepress@plos.org.

If we can help with anything else, please email us at plosone@plos.org. 

Thank you for submitting your work to PLOS ONE and supporting open access. 

Kind regards, 

PLOS ONE Editorial Office Staff

on behalf of

Dr. Hernâni Gerós 

Academic Editor

PLOS ONE
